# Shengmai San Ameliorates High-Glucose-Induced Calcium Homeostasis Imbalance via Improving Energy Metabolism in Neonatal Rat Cardiomyocytes

**DOI:** 10.3390/ph19040601

**Published:** 2026-04-08

**Authors:** Shixi Shang, Qu Zhai, Yuguo Huang, Junsong Yin, Jingju Wang, Xiaolu Shi

**Affiliations:** 1 Experimental Research Center, China Academy of Chinese Medical Sciences, Beijing 100070, China; shangshixi15@163.com (S.S.); huangyuguo127@163.com (Y.H.); yinjunsong1@163.com (J.Y.); wyctbzy@163.com (J.W.); 2 Institute of Executive Development, National Medical Products Administration, Beijing 100073, China; zhaiqu300@163.com

**Keywords:** Shengmai San, diabetic cardiomyopathy, calcium homeostasis, mitochondria, CaMKII, ROS

## Abstract

**Objective:** This study aims to investigate the protective effect of Shengmai San (SMS) against high-glucose (HG)-induced injury in neonatal rat ventricular myocytes (NRVMs) and to elucidate the underlying pharmacological molecular mechanisms. We hypothesize that SMS ameliorates HG-induced calcium homeostasis imbalance in NRVMs by improving mitochondrial energy metabolism disorder, and this protective effect is associated with the downregulation of oxidized and phosphorylated CaMKII expression to inhibit CaMKII signaling pathway overactivation. Herein, we verify this hypothesis by assessing mitochondrial function, calcium transients, sarcoplasmic reticulum (SR) calcium handling and CaMKII phosphorylation levels in NRVMs. **Methods:** First, ultra-high performance liquid chromatography–high resolution mass spectrometry was used to identify the chemical components of SMS to clarify its material basis. Primary NRVMs were then cultured under low-glucose (LG) or HG conditions, with 2% SMS-medicated serum (SMS-MS) as the experimental intervention, and NAC (ROS scavenger) and KN93 (CaMKII inhibitor) as positive controls. Following intervention, we sequentially detected key indicators corresponding to the proposed pathological pathway: intracellular reactive oxygen species (ROS) levels (oxidative stress), mitochondrial ROS, mitochondrial function indices including oxygen consumption rate (OCR) (energy metabolism), calcium transients and diastolic intracellular free calcium concentration (global calcium homeostasis), sarcoplasmic reticulum (SR) calcium leak (calcium handling disorder), and, finally, the phosphorylation, oxidation levels of CaMKII and RyR2 phosphorylation (Ser2814) (p-RyR2) (key regulatory pathway) via Western blot to systematically elucidate the mechanistic link between SMS intervention and HG-induced NRVM injury. **Results:** Quantitative analysis revealed that high-glucose (HG) induction significantly reduced calcium transient amplitude and prolonged the decay time constant (tau) in NRVMs at 72 h (*p* < 0.01 vs. LG), with these parameters normalizing by 120 h—an effect indicative of a compensatory adaptive response. The 2%SMS-MS markedly ameliorated HG-induced calcium transient abnormalities at 72 h (*p* < 0.01 vs. HG). Additionally, 2%SMS-MS significantly enhanced mitochondrial basal oxygen consumption rate, spare respiratory capacity, ATP production, and maximal respiration in HG-exposed NRVMs (*p* < 0.01 vs. HG). SMS also significantly reduced intracellular reactive oxygen species (ROS) levels (*p* < 0.01 vs. HG), mitochondrial ROS levels (*p* < 0.01 vs. HG), diastolic intracellular free calcium concentration (*p* < 0.01 vs. HG), and SR calcium leak (*p* < 0.05 vs. HG). Western blot analysis revealed that 2%SMS-MS intervention effectively downregulated the expression of oxidized CaMKII (Ox-CaMKII) (*p* < 0.01 vs. HG), phosphorylated CaMKII (p-CaMKII) (*p* < 0.01 vs. HG), and RyR2 phosphorylation (Ser2814) (*p* < 0.05 vs. HG), which may be the potential mechanism in maintaining calcium homeostasis in HG-induced NRVMs. **Conclusions:** This study suggests that SMS enhances mitochondrial energy metabolism and exerts a protective effect against high-glucose-induced calcium homeostasis imbalance in NRVMs, which supports our proposed hypothesis. Its potential mechanism indicates that the protective effects of SMS are associated with its ability to downregulate the expression of oxidized and phosphorylated CaMKII. These findings highlight SMS as a potential therapeutic candidate for alleviating HG-related myocardial injury and provide evidence for its application in the prevention of early diabetic cardiomyopathy.

## 1. Introduction

Excitation–contraction coupling (ECC) is fundamental to the heart’s pumping function, with intracellular calcium (Ca^2+^) homeostasis playing a central role in this process [1]. The calcium transient characterizes the rapid elevation and subsequent decline of cytosolic Ca^2+^ concentration following cardiomyocyte excitation; its amplitude and kinetic parameters directly correlate with myocardial contractility and relaxation capacity [2]. In the pathophysiology of diabetic cardiomyopathy (DCM), intracellular Ca^2+^ homeostasis is frequently impaired, characterized by a reduced amplitude of calcium transients, excessive depletion of SR calcium stores, and elevated diastolic cytosolic Ca^2+^ levels [3]. These aberrations in Ca^2+^ homeostasis not only compromise myocardial contractility but also predispose the heart to arrhythmias, thereby exacerbating the deterioration of cardiac function [4].

Previous studies have demonstrated that cardiomyocyte calcium signaling abnormalities occur in the early stages of diabetes. In early-stage type 1 diabetes mellitus (T1DM) rat models (3 weeks after streptozotocin [STZ] injection), cardiomyocytes exhibit impairments in systolic and diastolic calcium handling, primarily manifested as prolonged SR Ca^2+^ reuptake and reduced SERCA activity [5]. Notably, this loss of calcium handling capacity often precedes the onset of left ventricular systolic dysfunction. In contrast, the manifestations of impaired calcium handling in insulin resistance and early type 2 diabetes mellitus (T2DM) appear more complex and remain controversial. While impaired ventricular relaxation in some sucrose-fed and early T2DM models is associated with reduced expression of SERCA and RyR, significant changes in the expression of related genes in young Goto–Kakizaki (GK) and Zucker Diabetic Fatty (ZDF) rats lead to only a modest prolongation of contraction, with some studies even observing increased SERCA expression as a protective compensatory mechanism [6,7]. However, more investigations have revealed that despite the presence of such compensatory mechanisms, calcium homeostasis imbalance mediated by specific molecular pathways can still lead to severe pathological consequences. For instance, in a fructose-rich-diet (FRD)-induced prediabetes model, increased ROS production leads to the oxidative activation of CaMKII, which subsequently enhances SR Ca^2+^ leak via phosphorylation of RYR2 ryanodine receptor 2 (RyR2) at serine 2814. Although a compensatory increase in sarcoplasmic reticulum calcium ATPase (SERCA2a) activity helps maintain SR Ca^2+^ load, this disruption of calcium homeostasis is sufficient to trigger pro-arrhythmic calcium waves and arrhythmias in vivo [8]. Given that CaMKII-mediated calcium signaling abnormalities are already present in the early stages of diabetes, early intervention targeting this specific pathway may represent a key strategy for effectively delaying or ameliorating the subsequent onset and progression of DCM.

SMS is a classical traditional Chinese medicine formula composed of Ginseng, *Ophiopogon japonicus*, and *Schisandra Chinensis*. SMS possesses the properties of replenishing Qi, nourishing Yin, generating fluids, and checking sweating. It has been widely used in traditional medicine to treat cardiovascular conditions such as palpitations and shortness of breath caused by Qi and Yin deficiency. Previous studies have demonstrated that SMS and its pharmaceutical formulations (e.g., Shengmai injection) exert significant protective effects on the myocardium [9], including anti-myocardial ischemia–reperfusion injury and amelioration of heart failure [10,11,12,13]. Our preliminary experiments have demonstrated that SMS exerts cardioprotective effects in both type 1 and type 2 diabetic animal models, with mechanisms linked to the regulation of myocardial calcium homeostasis [14]. However, critical knowledge gaps remain in the current understanding of SMS’s cardioprotective effects in early hyperglycemic stress: First, existing evidence focuses primarily on pathological changes occurring after prolonged hyperglycemic exposure in in vivo diabetic models, and it remains unknown whether SMS exerts beneficial effects on cardiomyocytes under short-term high-glucose conditions—an early stage of hyperglycemic stress that precedes overt diabetic pathological remodeling. Second, robust endogenous compensatory mechanisms in in vivo pathological environments often obscure the early functional phenotypic changes in cardiomyocytes and the direct regulatory effects of SMS on cardiomyocyte intrinsic metabolism and calcium signaling. Third, the mechanistic link between SMS-mediated improvement of mitochondrial energy metabolism and the inhibition of CaMKII overactivation in early high-glucose-induced calcium homeostasis imbalance has not been systematically elucidated and experimentally verified in a pure cardiomyocyte model. To address these unresolved gaps, the present study utilized a cultured neonatal rat ventricular myocyte (NRVM) model exposed to a high-glucose medium—a cell model that eliminates systemic compensatory interference and allows for the direct observation of early cardiomyocyte functional changes in response to short-term hyperglycemic stress. This approach enabled us to specifically investigate the following: (1) whether SMS can intervene in high-glucose-induced calcium transient impairment and calcium homeostasis imbalance in the early stage of elevated blood glucose, before endogenous cellular compensation is initiated; (2) the direct effect of SMS on mitochondrial energy metabolism in HG-exposed NRVMs, and its correlation with the reduction in intracellular oxidative stress; and (3) the mechanistic connection between SMS-mediated regulation of mitochondrial function and the inhibition of CaMKII oxidation/phosphorylation, and how this link modulates SR calcium handling to maintain calcium homeostasis.

To capture these early, subtle functional changes, we utilized cultured NRVMs. This model is ideally suited for investigating early hyperglycemic stress because NRVMs exhibit robust survival in culture and distinct excitation–contraction coupling properties, allowing for the detection of early-stage metabolic dysfunction and transient calcium handling aberrations that might be obscured by the complex systemic environment or age-related structural changes present in adult in vivo models.

This study, for the first time, clarifies the time-dependent characteristics of high-glucose-induced calcium transient dysfunction in NRVMs; further, it establishes a direct molecular link between SMS-mediated mitochondrial energy metabolism improvement and CaMKII-dependent calcium homeostasis regulation in the early stage of hyperglycemic stress. Our findings are intended to provide novel experimental evidence and a theoretical foundation for the application of SMS in the prevention and treatment of DCM, especially for intervening in the early stages of diabetes when cardiomyocytes are exposed to a hyperglycemic microenvironment, and to further expand the understanding of the multi-target regulatory mechanisms of classic TCM formulas in cardiovascular metabolic diseases.

## 2. Result

### 2.1. Analysis of Chemical Components of SMS-MS

**Figure 1:**
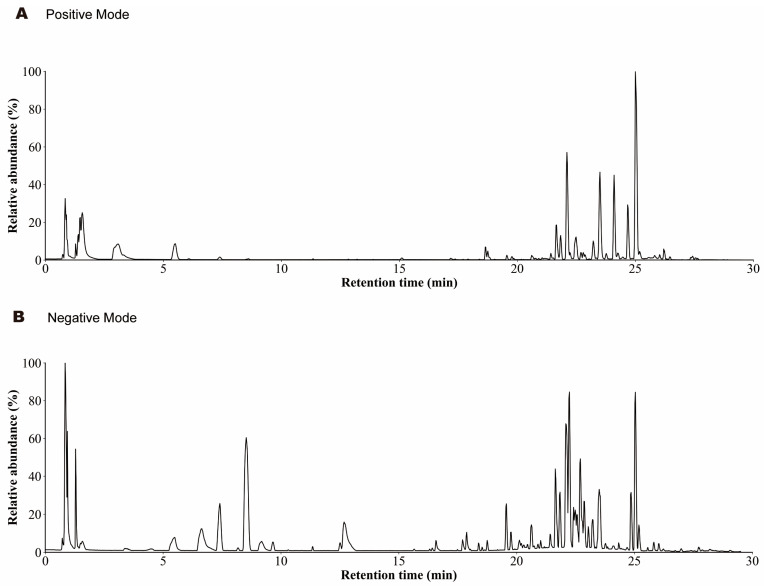
High-resolution extracted ion chromatograms of SMS-MS acquired using ultra-high performance liquid chromatography–hybrid quadrupole–Orbitrap high-resolution mass spectrometry (UHPLC-Q-Orbitrap HRMS). (**A**) Base peak intensity chromatogram of SMS-MS in positive ion mode. (**B**) Base peak intensity chromatogram of SMS-MS in negative ion mode.

To detect and identify the prototype components in SMS-MS, we investigated its chemical composition using a UHPLC-Q-Orbitrap HRMS platform. The SMS-MS samples were scanned using positive and negative ion modes (Figure 1A,B). As shown in Table S1, the detected compounds of SMS-MS covered multiple categories, primarily including terpenoids, the detected compounds of SMS-MS covered multiple categories, primarily including terpenoids (e.g., ginsenosides) and lignans (e.g., Schisandra lignans). Experimental data indicated that components derived from Ginseng were primarily dammarane-type tetracyclic triterpenoid saponins, with Ginsenoside Rb1 (*m*/*z* 1109.6130, retention time 17.72 min), Ginsenoside Rd (*m*/*z* 991.5471, retention time 18.53 min), Ginsenoside Rg3 (*m*/*z* 829.4988, retention time 20.47 min), and Ginsenoside Rg5 (*m*/*z* 767.4924, retention time 22.07 min) as the representative identified components; these saponins exhibited high chromatographic response intensities, constituting the primary pharmacological material basis of SMS. Components derived from *Schisandra chinensis* were characterized by lignans, mainly including Schisandrol A (*m*/*z* 433.2215, retention time 20.02 min), Gomisin D (*m*/*z* 531.2217, retention time 20.26 min), Schisanhenol (*m*/*z* 403.2105, retention time 22.38 min), and Schisandrin B (*m*/*z* 401.1952, retention time 23.84 min), among which Gomisin D showed the highest peak area (417728) and was the most abundant lignan component in the medicated serum. All identified components were prototype constituents without obvious metabolic transformation in the in vivo serum environment. It should be noted that, in this study, only the qualitative identification of the active metabolites and prototype components in SMS medicated serum was completed via UHPLC-Q-Orbitrap HRMS, and the quantitative analysis of the content of each component was not carried out due to the lack of corresponding standard products for partial components and the complexity of the serum matrix. This is a limitation of the current study, and the relative content and absolute concentration of each active component in the medicated serum, as well as the dose–effect relationship between the components and the cardioprotective effect of SMS, need to be further verified by establishing a complete quantitative analysis method in subsequent research.

### 2.2. Network Pharmacology Analysis of SMS-MS

#### 2.2.1. Screening of Potential Targets for SMS-MS Compounds

**Figure 2:**
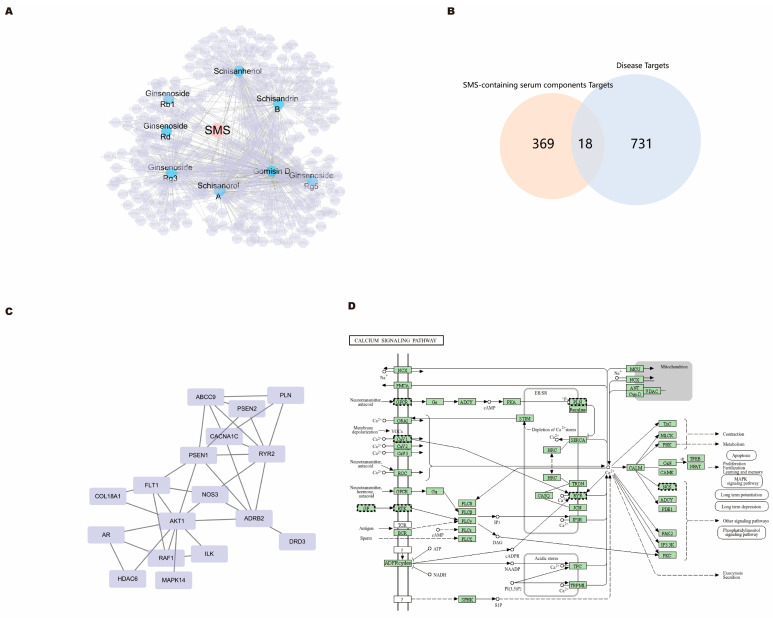
Network pharmacology analysis reveals that SMS-MS can synergistically improve high-glucose-induced calcium homeostasis imbalance in cardiomyocytes. (**A**) Target network of SMS-MS compounds. (**B**) Venn diagram of SMS-disease intersection targets. (**C**) Protein–protein interaction (PPI) network of core targets. (**D**) KEGG pathway map (https://www.genome.jp/kegg/) accessed on 17 March 2026.

Database searches identified 9 targets for Ginsenoside Rb1, 13 targets for Ginsenoside Rd, 108 targets for Ginsenoside Rg3, 105 targets for Ginsenoside Rg5, 106 targets for Schisanhenol, 110 targets for Schisandrol A, 105 targets for Schisandrin B, and 110 targets for Gomisin D. After removing duplicates, 387 targets were finally obtained (Figure 2A), indicating that SMS serum compounds exert therapeutic effects through multiple targets.

#### 2.2.2. Acquisition of Disease Targets and Identification of Intersection Targets

Database searches retrieved 818 targets for “diabetic cardiomyopathy,” 185 targets for “myocardial dysfunction”, 36 targets for “systolic dysfunction”, and 183 targets for “diastolic dysfunction”. After removing duplicates, 749 disease targets were finally obtained. Venny 2.1 identified 18 intersection targets between SMS and disease (Figure 2B).

#### 2.2.3. Construction of PPI Network and and Core Target Analysis

The 18 intersection targets were imported into the STRING database, and the TSV file was downloaded and imported into Cytoscape 3.7.1. Due to insufficient protein–protein interaction data for TNNI3K in the STRING database, it was automatically filtered by the STRING platform, and 17 targets were finally included in the PPI network analysis. Network topology parameters were analyzed using the Network Analyzer function in Cytoscape, and the PPI network was constructed (Figure 2C).

#### 2.2.4. Enrichment Analysis Results

The 18 core targets were imported into the DAVID platform, yielding 88 BP-related terms, 20 CC-related terms, 20 MF-related terms in GO analysis, and 38 KEGG-related pathways (Tables S2–S5).

##### GO_BP Enrichment Analysis Results

BP analysis showed that biological processes are primarily involved in calcium ion homeostasis regulation, myocardial contraction regulation, MAPK signaling, vascular function regulation, and negative regulation of apoptosis. At the calcium ion level, targets were significantly enriched in excitation–contraction coupling-related processes in cardiomyocytes: “intracellular calcium ion homeostasis” and “regulation of cardiac muscle contraction by regulation of the release of sequestered calcium ion”, which precisely corresponds to the central role of sarcoplasmic reticulum (SR) calcium release channel RyR2-mediated calcium release in myocardial contraction regulation. Meanwhile, targets were also significantly enriched in “calcium ion transport”, “calcium ion transmembrane import into cytosol”, and “regulation of cytosolic calcium ion concentration”. At the functional effect level, targets were significantly enriched in “regulation of heart contraction”, “cardiac conduction”, and “regulation of ventricular cardiac muscle cell action potential”. In addition, targets were significantly enriched in “negative regulation of apoptotic process”, suggesting that Shengmai San may delay myocardial injury progression by inhibiting apoptotic signaling.

##### GO_CC Enrichment Analysis Results

CC analysis showed that cellular components were mainly distributed in sarcolemma, sarcoplasmic reticulum, sarcomere, mitochondria, and various protein complexes. These structures play key roles in calcium signal transduction, processing, and energy supply in cardiomyocytes. In the calcium signal transduction stage, targets were significantly enriched in the sarcolemma and plasma membrane. As a critical interface for extracellular-to-intracellular calcium signal transduction, the sarcolemma harbors L-type calcium channels, sodium–calcium exchangers, and various G protein-coupled receptors, which are important structures for calcium signal initiation. In the calcium signal processing stage, targets were significantly enriched in sarcoplasmic reticulum membrane, sarcoplasmic reticulum, and sarcomere. Sarcoplasmic reticulum is the central organelle for calcium storage, release, and reuptake in cardiomyocytes, with both RyR2 calcium release channel and SERCA2a calcium pump localized here; sarcomere is the basic functional unit of myocardial contraction, and its contractile activity directly depends on calcium ion-induced myofilament sliding. In the energy supply stage, targets were significantly enriched in mitochondria. Mitochondria are not only the main source of ATP in cardiomyocytes but also participate in calcium buffering and reactive oxygen species generation, with their functional status closely related to calcium homeostasis.

##### GO_MF Enrichment Analysis

MF analysis showed that molecular functions mainly involved calmodulin binding, calcium channel activity, protein kinase activity, ATPase binding, etc. Calmodulin is a key sensor for intracellular calcium signals; calcium channel activity directly corresponds to L-type calcium channels and RyR2-mediated calcium ion transmembrane or trans-organelle transport, which is the molecular basis for excitation–contraction coupling in cardiomyocytes. Protein kinase activity, protein serine kinase activity, and protein serine/threonine kinase activity involve the catalytic functions of key signaling molecules such as CaMKII, PKA, PKC, and MAPK. In terms of energy metabolism-related functions, targets were significantly enriched in ATP binding and nucleotide binding. ATP is the energy substrate for calcium pumps (SERCA2a) and various kinases, and is closely related to calcium reuptake regulation.

##### KEGG Pathway Enrichment Analysis

KEGG pathway enrichment analysis primarily involved calcium signaling pathway, adrenergic signaling in cardiomyocytes, cGMP-PKG signaling, cAMP signaling, and MAPK signaling pathways, which are closely related to myocardial calcium homeostasis regulation, presenting clear signal cascade characteristics. At the core calcium signaling pathway level, targets were significantly enriched in the “calcium signaling pathway” (Figure 2D), which encompasses L-type calcium channels, RyR2, calmodulin, and CaMKII, directly regulating calcium ion influx, release, and downstream effects. In addition, targets were also enriched in the “MAPK signaling pathway.” Moreover, the MAPK pathway has extensive bidirectional interactions with calcium signaling. At the disease-related pathway level, targets were significantly enriched in “diabetic cardiomyopathy” and the “AGE-RAGE signaling pathway in diabetic complications,” both of which are directly related to the disease background of this study.

### 2.3. SMS-MS Corrects High-Glucose-Induced Abnormal Calcium Transients in Neonatal Rat Cardiomyocytes

**Figure 3:**
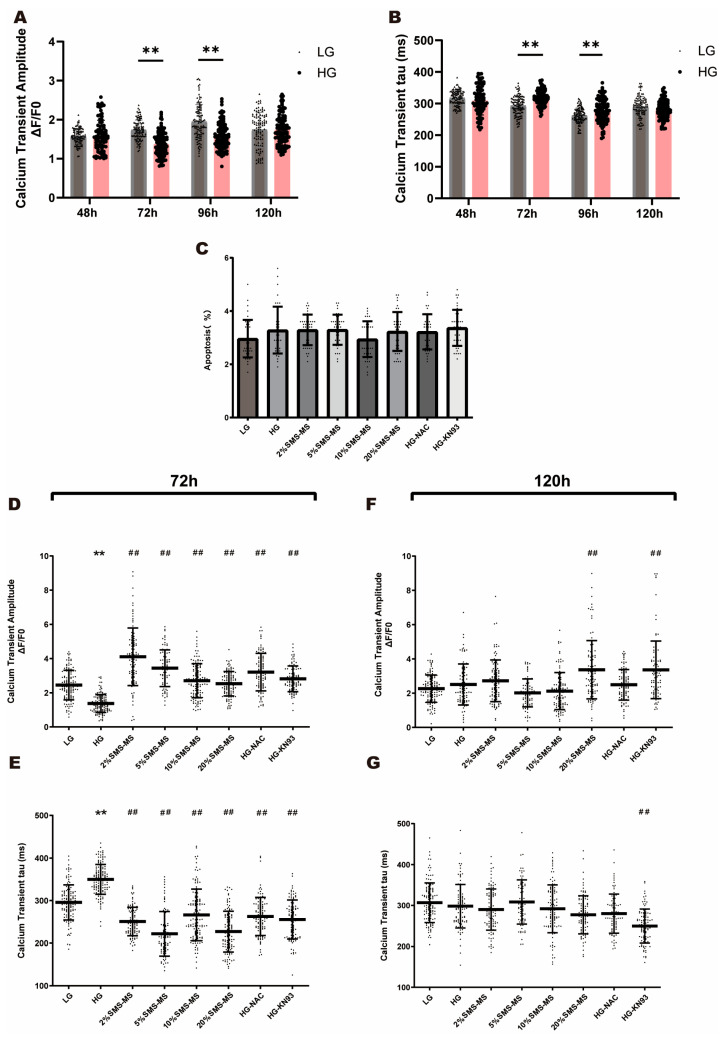
SMS-MS enhances calcium influx in high-glucose-induced injured cardiomyocytes. (**A**) Amplitude of calcium transient (ΔF/F_0_) in cardiomyocytes of the LG and HG groups at 48, 72, 96, and 120 h (*n* = 105–115 cells). (**B**) Tau values of calcium transients in cardiomyocytes of the LG and HG groups at 48, 72, 96, and 120 h (*n* = 105–115 cells). (**C**) TUNEL fluorescence intensity in cardiomyocytes of each group after 120 h of high-glucose induction (indicating the apoptotic rate; *n* = 41 cells). (**D**) Amplitude of calcium transient (ΔF/F_0_) in cardiomyocytes of each group after 72 h of high-glucose induction (*n* = 84–140 cells). (**E**) Tau values of calcium transients in cardiomyocytes of each group after 72 h of high-glucose induction (*n* = 81–125 cells). (**F**) Amplitude of calcium transient (ΔF/F_0_) in cardiomyocytes of each group after 120 h of high-glucose induction (*n* = 84–140 cells). (**G**) Tau values of calcium transients in cardiomyocytes of each group after 120 h of high-glucose induction (*n* = 81–125 cells). Data are expressed as mean ± SD from 6 independent biological replicates (cardiomyocyte isolations), with each data point representing a single-cell technical replicate (indicated by “*n*”). Statistical significance was assessed by one-way analysis of variance (ANOVA). ** *p* < 0.01 vs. LG; ## *p* < 0.01 vs. HG.

Calcium transient refers to the rapid fluctuation of cytosolic calcium ion concentration during the depolarization of cardiomyocytes and plays a key role in their contractile function. First, we assessed 1 Hz electrical pacing-induced calcium transient characteristics in low-glucose (LG) and high-glucose (HG) group cardiomyocytes, which were loaded with the calcium-sensitive fluorescent dye Fluo-4 and subjected to detection at 48, 72, 96 and 120 h post-induction. The results showed that in the early stage of HG induction, specifically 72 h after HG induction of NRVMs, the amplitude of calcium transients (F/F_0_) was significantly reduced (Figure 3A), while the calcium transient decay time constant (tau) was significantly prolonged compared with the LG group (Figure 3B). However, after 120 h of HG induction, both the amplitude and tau values recovered to levels close to the LG group (Figure 3A,B), indicating a spontaneous reversal of calcium handling dysfunction in NRVMs under prolonged high-glucose exposure. This time-dependent recovery was accompanied by no detectable apoptotic changes in NRVMs (Figure 3C), suggesting that the calcium dysfunction at 120 h was a reversible functional injury rather than irreversible structural damage, and that the recovery at 120 h was likely mediated by intrinsic compensatory mechanisms of neonatal cardiomyocytes rather than cell renewal or replacement. Therefore, we focused on the effects of SMS on calcium transients at 72 h and 120 h of HG induction. At 72 h, all medication groups significantly restored the reduced calcium transient amplitude and the elevated calcium transient decay constant. Notably, the 2%SMS-MS showed superior efficacy in restoring the HG-induced reduction in calcium transient amplitude and the prolongation of the decay constant (Figure 3D,E). At 120 h, SMS shows some improvement, but it is less significant than that at 72 h (Figure 3F,G), which is consistent with the spontaneous compensatory recovery of calcium function in NRVMs at this time point. These results suggest that SMS can restore calcium signal processing in HG-injured cardiomyocytes, which may be one of the important mechanisms underlying its improvement of myocardial function, especially before the initiation of endogenous compensatory changes (72 h).

### 2.4. SMS Ameliorates High-Glucose-Induced Mitochondrial Energy Metabolism Disorders in Neonatal Rat Cardiomyocytes

**Figure 4:**
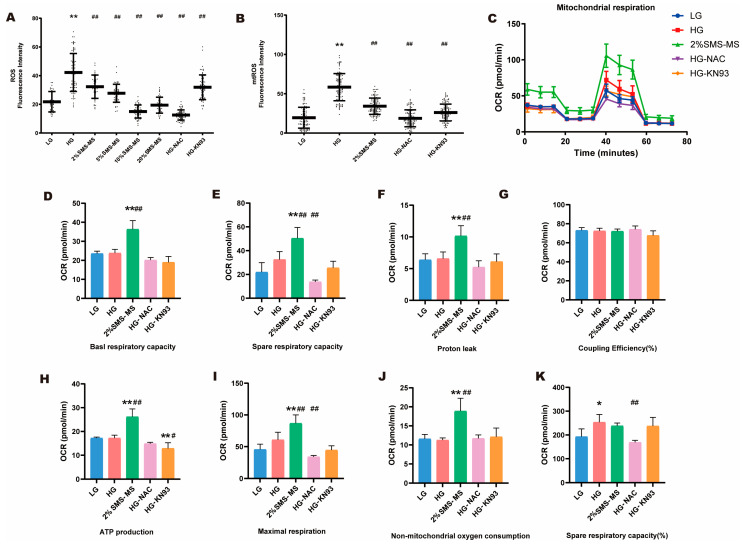
SMS-MS enhances mitochondrial energy metabolism in high-glucose-injured cardiomyocytes. (**A**) ROS fluorescence intensity in cardiomyocytes of each group after 72 h of high-glucose induction (*n* = 34–64 cells). (**B**) Mitochondrial ROS (mtROS) fluorescence intensity in cardiomyocytes of each group after 72 h of high-glucose induction (*n* = 100–120 cells). Mitochondrial energy metabolism was assessed using the Seahorse XF mitochondrial stress test. (**C**) Mitochondrial respiratory curves of cardiomyocytes in each group (*n* = 45). The following parameters were calculated from these curves: (**D**) basal respiration; (**E**) spare respiratory capacity; (**F**) proton leak; (**G**) coupling efficiency (%); (**H**) ATP production; (**I**) maximal respiration; (**J**) non-mitochondrial oxygen consumption; (**K**) spare respiratory capacity (%). Data are expressed as mean ± SD, and statistical significance was assessed by one-way analysis of variance (ANOVA). * *p* < 0.05, ** *p* < 0.01 vs. LG; ## *p* < 0.01 vs. HG.

Oxidative stress is an early core event in HG-induced cardiomyocyte injury. We used the DCFH-DA probe to detect intracellular ROS levels. The results showed that after 72 h of HG culture, ROS production was higher in the HG group compared with the LG group. All medication groups could significantly reduce the HG-induced increase in ROS (Figure 4A). The effects of 2%SMS-MS in reducing ROS products exhibited a certain dose-dependence. This demonstrates that SMS possesses potent antioxidant capacity and can effectively reduce excess ROS induced by high glucose. To further identify the mtROS—the main subcellular source of ROS in HG-induced cardiomyocyte injury—we performed targeted detection using the mitochondrial-specific fluorescent probe MitoSOX Red. The results showed that HG exposure for 72 h markedly elevated the mtROS level in NRVMs, which was consistent with the mitochondrial metabolic stress observed in the HG group (Figure 4B). The 2%SMS-MS intervention significantly downregulated the HG-induced mtROS overproduction, and its inhibitory effect on mtROS was comparable to that of the positive control NAC and KN93, indicating that SMS can specifically target mitochondrial oxidative stress and reduce the excessive generation of mtROS in HG-exposed NRVMs (Figure 4B).

To investigate the underlying mechanism by which SMS mitigates ROS production, we evaluated the effect of SMS on energy metabolism in cardiomyocytes under high-glucose conditions, and we performed a Seahorse mitochondrial stress test (Figure 4C). Notably, basal mitochondrial oxygen consumption rate (OCR) in the HG group was not significantly reduced compared with the LG group, a finding that reflects an early metabolic stress adaptation response of neonatal rat cardiomyocytes (NRVMs) to acute hyperglycemic exposure rather than severe, irreversible mitochondrial structural or functional damage. Despite the absence of overt basal respiration impairment, the HG group exhibited characteristic signs of mitochondrial metabolic stress: the spare respiratory capacity (%) was significantly increased relative to the LG group (Figure 4K), a compensatory upregulation that represents the mitochondria’s attempt to maintain energy supply by mobilizing reserve respiratory capacity in response to HG-induced metabolic disturbance. Additionally, the HG-induced ROS overproduction (Figure 4A,B) further confirmed mitochondrial oxidative stress, a key manifestation of early mitochondrial dysfunction in the setting of metabolic adaptation. In contrast to the HG group’s adaptive metabolic state, the 2%SMS-MS group (after 72 h of HG induction) exhibited significantly increased mitochondrial basal metabolic rate (basal OCR) (Figure 4D), spare respiratory capacity (Figure 4E), proton leak (Figure 4F) and actual ATP synthesis capacity (ATP production) (Figure 4H). Additionally, the increased maximal respiration rate in the 2%SMS-MS group indicated that the cells possessed greater potential to resist injury caused by stressors such as high glucose or hypoxia (Figure 4I). Meanwhile, ATP production and non-mitochondrial oxygen consumption were both increased in the 2%SMS-MS group (Figure 4H,J), indicating an active energy metabolic state. In contrast, the basal respiration rate and ATP production rate in the NAC and KN93 groups showed no significant differences compared to the LG and HG groups. Compared with the LG group, the spare respiratory capacity (%) in the HG group was significantly increased. The 2%SMS-MS group partially alleviated the increase caused by high glucose, but there was no significant difference (Figure 4K), which reflected a normalization of the mitochondrial compensatory state: by enhancing basal mitochondrial metabolic function and ATP synthesis capacity, SMS reduced the cellular need for sustained compensatory mobilization of spare respiratory capacity, thus restoring mitochondrial respiration to a more physiological homeostatic state where basal function is robust and reserve capacity is maintained at a physiological level. There were no significant differences in coupling efficiency (%) among the various groups (Figure 4G).

### 2.5. SMS-MS Reduces SR Calcium Leak by Inhibiting CaMKII Phosphorylation

#### 2.5.1. SMS-MS Alleviates High-Glucose-Induced Intracellular Calcium Overload in Neonatal Cardiomyocytes

**Figure 5:**
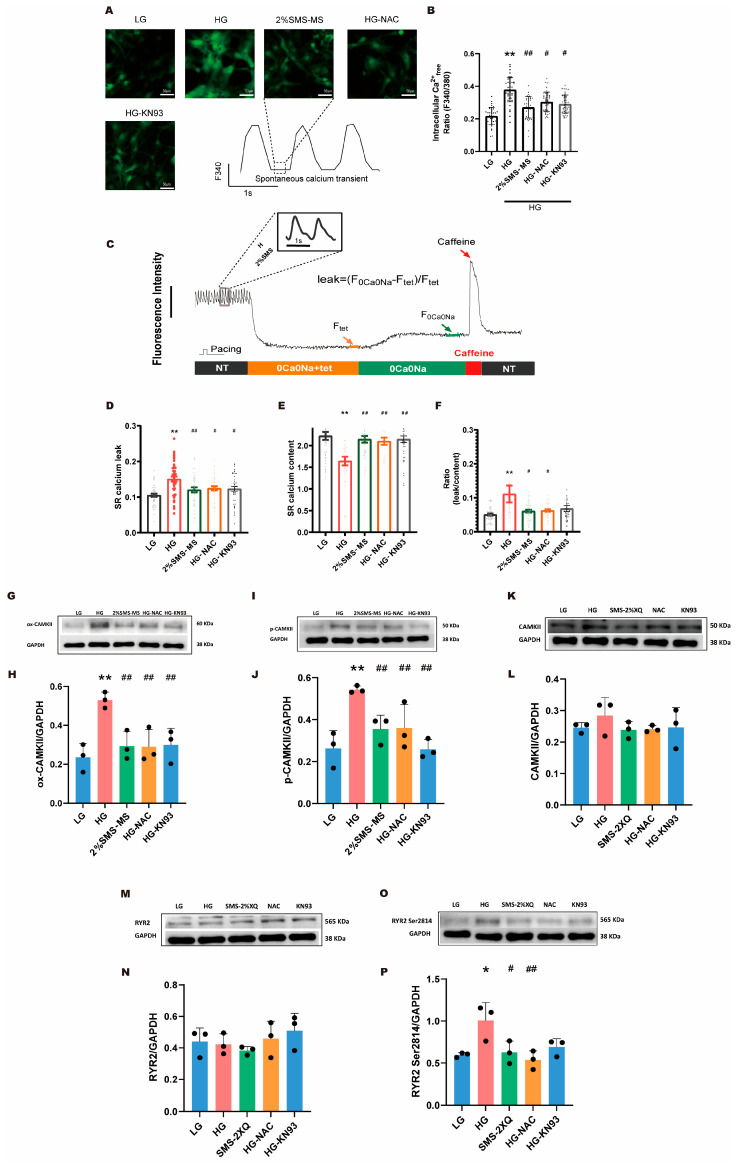
SMS-MS interventions ameliorated high-glucose-induced SR calcium handling dysfunction in neonatal cardiomyocytes. (**A**) Demo calcium image of the Fura-2 dye in 340 nm fluorescence channel. (**B**) The intracellular free calcium concentration ratio (F340/380) in cardiomyocytes (*n* = 37–46 cells). (**C**) Typical Fluo-4 fluorescence intensity changes follow in the SR leak experimental protocol. (**D**) Quantification of SR calcium leak (*n* = 45–48 cells). (**E**) Quantification of SR calcium content (assessed by caffeine-induced calcium release amplitude) (*n* = 45–48 cells). (**F**) Normalized SR calcium leak ratio (SR calcium leak/SR calcium content) to assess relative leak severity (*n* = 45–48 cells). Data are expressed as mean ± SD from 6 independent biological replicates (cardiomyocyte isolations), with each data point representing a single-cell technical replicate (indicated by “*n*”). (**G**) Western blot analysis of ox-CAMKII expression in cardiomyocytes after respective treatments for 72 h. (**H**) Quantitative analysis of ox-CAMKII protein levels normalized to GAPDH (*n* = 3). (**I**) Western blot analysis of p-CAMKII expression in cardiomyocytes after respective treatments for 72 h. (**J**) Quantitative analysis of p-CAMKII protein levels normalized to GAPDH (*n* = 3). (**K**) Western blot analysis of CAMKII expression in cardiomyocytes after respective treatments for 72 h. (**L**) Quantitative analysis of CAMKII protein levels normalized to GAPDH (*n* = 3). (**M**) Western blot analysis of RyR2 expression in cardiomyocytes after respective treatments for 72 h. (**N**) Quantitative analysis of RyR2 protein levels normalized to GAPDH (*n* = 3). (**O**) Western blot analysis of p-RyR2 expression in cardiomyocytes after respective treatments for 72 h. (**P**) Quantitative analysis of p-RyR2 protein levels normalized to GAPDH (*n* = 3). Data shown are individual values of mean ± SD, and statistical significance was assessed by one-way analysis of variance (ANOVA). * *p* < 0.05, ** *p* < 0.01 vs. LG; # *p* < 0.05, ## *p* < 0.01 vs. HG. Scale bars: 50 μm (**A**).

To investigate the impact of high glucose (HG) on diastolic calcium homeostasis, we first quantified the intracellular free calcium concentration in neonatal cardiomyocytes under experimental conditions (assessed as the F340/380 fluorescence ratio; Figure 5A,B). Notably, in evaluating diastolic calcium—distinct from calcium transient detection—we employed the ratiometric fluorescent dye Fura-2, which enables more accurate reflection of the diastolic calcium concentration. Although neonatal rat cardiomyocytes exhibit spontaneous calcium transients, their spontaneous calcium release rate was less than 1 Hz at room temperature. Compared with calcium transients induced by regular 1 Hz electrical stimulation, this slower spontaneous calcium release from SR allowed a longer time for diastolic calcium to return to a relatively low level, thereby better reflecting the true physiological calcium level. For this reason, we used the steady calcium fluorescence intensity ratio (F340/F380) of the cells to represent the cytoplasmic calcium level. Figure 5A shows a typical fluorescence image obtained under a 340 nm excitation light signal.

Compared with the low-glucose (LG) group, exposure to HG led to a marked elevation in diastolic calcium levels, confirming that HG triggers diastolic calcium overload—a hallmark of myocardial dysfunction. When cardiomyocytes were treated with 2%SMS-MS, NAC, and KN93, all three agents attenuated HG-induced calcium elevation, with 2%SMS-MS demonstrating the most robust corrective effects (Figure 5B).

#### 2.5.2. SMS-MS Reduces SR Calcium Leak and Calcium Content Impaired by High Glucose

To dissect the underlying mechanisms of HG-induced calcium dysregulation, we performed dynamic fluorescence recordings to track SR calcium handling throughout the experimental protocol (Figure 5C). Building on the dynamic recordings, we systematically quantified SR calcium leak and SR calcium content to clarify how HG disrupts SR calcium homeostasis. Relative to the LG group, the HG group exhibited a significant increase in SR calcium leak (Figure 5D), indicating enhanced abnormal calcium efflux from the SR. High glucose also impairs SR calcium leak and calcium content (Figure 5E), reflecting impaired calcium storage capacity of the SR. Treatment with 2%SMS-MS, NAC or KN93 effectively reduced this HG-induced calcium leak and successfully restored SR calcium content toward LG levels.

To eliminate the confounding influence of reduced SR calcium content on leak measurements, we calculated the normalized leak ratio (SR calcium leak/SR calcium content) to assess the relative severity of SR calcium dysregulation (Figure 5F). The HG group displayed a marked increase in this normalized ratio, confirming that HG elevates the relative risk of SR calcium leak independent of changes in total calcium storage. Notably, 2%SMS-MS or NAC significantly reduced this normalized ratio, demonstrating their ability to normalize SR calcium handling, with 2%SMS-MS demonstrating the most robust corrective effects.

Taken together, these findings demonstrate that HG exposure disrupts multiple facets of calcium homeostasis in neonatal cardiomyocytes, including diastolic calcium overload, increased SR calcium leak, reduced SR calcium content, and an elevated relative risk of SR calcium leakage. Pharmacological interventions with 2%SMS-MS, NAC or KN93 effectively ameliorated these HG-induced calcium dysregulations, highlighting their potential as therapeutic candidates for mitigating HG-related myocardial injury.

#### 2.5.3. SMS-MS Inhibits the Expression of Oxidation and Phosphorylation of CaMKII

To explore the deep mechanism by which SMS improves cellular calcium homeostasis, we detected the phosphorylation level of the key signaling molecule CaMKII. After 72 h of HG culture, Western blot results showed that the total CaMKII protein expression (Figure 5K,L) and total RyR2 (Figure 5M,N) remained stable across all groups. Compared with the LG group, the expression of Ox-CaMKII (Figure 5G,H), p-CaMKII (Figure 5I,J), and p-RyR2 was upregulated in the HG group. The 2% SMS-MS group was able to reduce the upregulation of Ox-CaMKII, p-CaMKII, and p-RyR2 expression. These findings establish an associative link between CaMKII oxidation/phosphorylation and HG-induced SR calcium leak and calcium homeostasis imbalance in NRVMs; combined with the protective effects of the CaMKII pharmacological inhibitor KN93 on HG-induced calcium dysregulation (Figure 5B,D–F), they support a role for CaMKII in mediating HG-induced myocardial calcium dysfunction. It is important to note that the mechanistic link between CaMKII activity and the calcium regulatory effects of SMS is based on these pharmacological inhibition data and molecular associations, and has not been validated by genetic manipulation of CaMKII in the present study. A critical downstream pathological effect of CaMKII overactivation is the phosphorylation of RyR2, leading to SR calcium leak.

## 3. Discussion

DCM is a major cause of heart failure in patients with diabetes. One of its core pathological bases is the disorder of cardiomyocyte excitation–contraction coupling [15,16] and the disruption of calcium homeostasis, characterized by a decrease in calcium transient amplitude, depletion of the SR calcium store, and elevated diastolic cytosolic free Ca^2+^ [17]. These alterations not only weaken myocardial contractility but also increase the risk of arrhythmias, thereby accelerating the deterioration of cardiac function [18]. High-glucose-induced mitochondrial dysfunction [19,20] and excessive generation of ROS [21] are widely recognized as key upstream events triggering these changes. Reduced mitochondrial ATP production limits energy-consuming processes such as SERCA activity and further damages proteins and membrane structures via excessive ROS [22], forming a vicious cycle of “energy metabolism disorder–ROS–calcium homeostasis imbalance.”

The major bioactive constituents of SMS-MS exhibit complementary antioxidant, mitochondrial-protective and Ca^2+^-regulating activities that are mechanistically relevant to diabetic cardiomyopathy. Ginsenoside Rb1 improves diabetic cardiomyopathy by reducing sarcoplasmic reticulum Ca^2+^ leak through overactivated RyR2, enhancing SERCA2a-mediated Ca^2+^ reuptake, and correcting O-GlcNAcylation of Ca^2+^-handling proteins, thereby restoring intracellular Ca^2+^ homeostasis and myocardial energy metabolism [23]. Rb1 additionally mitigates oxidative/carbonyl stress, increases SOD, CAT and Gpx activities, and lowers MDA levels in diabetic hearts, indicating robust antioxidant protection [24,25]. Ginsenoside Rd attenuates myocardial ischemic injury and heart failure by promoting mitochondrial biogenesis (upregulating PGC-1α, NRF1/2 and TFAM, increasing mtDNA and mitochondrial membrane potential) and inhibiting WNT5A/Ca^2+^–CaMKII signaling to reduce Ca^2+^ overload and ROS production [26,27]. Ginsenoside Rg3 protects against diabetic cardiomyopathy via direct activation of PPAR-γ, enhancing adiponectin secretion and signaling, improving systemic glucose–lipid metabolism, and thereby alleviating structural and functional cardiac injury; Rg3 also suppresses NLRP3 inflammasome activation and oxidative stress through modulation of the SIRT1/NF-κB pathway in hypertrophic myocardium [28,29]. Schisandra lignans such as Schisandrin A and Schisandrin B are classical antioxidants that improve cardiac function and reduce hypertrophy, fibrosis, inflammation and oxidative stress in diabetic cardiomyopathy models, potentially via inhibition of complement cascade activation, although direct regulation of CaMKII- or RyR2-dependent sarcoplasmic reticulum Ca^2+^ leak has not been conclusively demonstrated [30]. Collectively, these findings suggest that the holistic cardioprotective efficacy of SMS-MS against high-glucose-induced Ca^2+^ homeostasis imbalance stems from coordinated reductions in oxidative stress, improved mitochondrial metabolism, and modulated Ca^2+^-handling pathways, with the previously validated properties of our UHPLC-Q-Orbitrap HRMS-identified components providing a robust theoretical foundation for this mechanism.

The network pharmacology analysis of this study reveals that the major active components in SMS serum (such as Ginsenoside Rb1, Rd, Rg3, Rg5, Schisandrol A, Schisandrin B, etc.) can synergistically improve high-glucose-induced calcium homeostasis imbalance in cardiomyocytes through multiple targets and pathways, providing a systematic mechanistic interpretation for the experimental observations that SMS ameliorates calcium transients, reduces diastolic calcium concentration, and decreases SR calcium leak.

Network pharmacology GO enrichment analysis showed that intersection targets were significantly enriched in biological processes such as “intracellular calcium ion homeostasis” and “regulation of cardiac muscle contraction by regulation of the release of sequestered calcium ion,” with cellular components mainly distributed in sarcolemma, sarcoplasmic reticulum membrane, and sarcomere, and molecular functions involving calmodulin binding and calcium channel activity. This analysis result is highly consistent with our experimental findings: SMS significantly reduced Ox-CaMKII and p-CaMKII expression levels, inhibited RyR2-mediated SR calcium leak, and restored SR calcium content and calcium transient amplitude. Network pharmacology enrichment analysis suggests that SR calcium release channel (RyR2) and calcium reuptake pump (SERCA2a) are key molecules maintaining SR calcium homeostasis, and their dysfunction directly leads to diastolic calcium overload and contractile dysfunction. Our pharmacological evidence indicates that SMS may modulate this pathological process through inhibition of CaMKII overactivation, which is associated with a reduction in RyR2-mediated SR calcium leak.

KEGG pathway enrichment analysis showed that SMS active component targets were significantly enriched in the calcium signaling pathway, MAPK signaling pathway, cGMP-PKG and cAMP signaling pathways, as well as the AGE-RAGE signaling pathway related to diabetic cardiomyopathy. Notably, the MAPK pathway has extensive bidirectional interactions with calcium signaling, and excessive activation of the MAPK pathway can exacerbate oxidative stress and cell damage, which is consistent with our experimental results that SMS reduced ROS levels and improved mitochondrial energy metabolism. The “negative regulation of apoptotic process” predicted by network pharmacology was also supported by experiments: within the 72 h high-glucose exposure window, TUNEL detection showed that cells did not enter the apoptotic state, while SMS intervention at this critical period effectively blocked the progression from functional disorder to structural damage.

Among the 18 SMS-disease intersection targets identified by network pharmacology analysis, multiple targets participate in myocardial energy metabolism regulation, including ATP-binding and nucleotide-binding functions. This is consistent with Seahorse mitochondrial stress test results: SMS significantly enhanced mitochondrial basal respiration rate, ATP production capacity, and maximal respiration capacity in cardiomyocytes, providing sufficient energy substrates for calcium-handling proteins such as SERCA2a. In contrast, although the single antioxidant NAC or CaMKII inhibitor KN93 could alleviate oxidative stress and calcium homeostasis disorders, they failed to simultaneously enhance mitochondrial energy metabolism like SMS, demonstrating the integrative advantages of multi-component synergistic regulation of traditional Chinese medicine compound formulas.

In addition, biological processes involved in network pharmacology enrichment analysis, such as “cardiac conduction” and “regulation of ventricular cardiac muscle cell action potential,” provide a potential molecular basis for SMS improvement of calcium transient kinetic parameters (such as shortening decay time constant tau). SMS regulates multiple key signaling pathways, breaking the vicious cycle of “energy metabolism disorder–excessive ROS generation–CaMKII overactivation–calcium homeostasis imbalance” under a high-glucose environment, and elucidates the molecular network mechanism of its protective effect on cardiomyocyte calcium homeostasis from a systems biology perspective.

Our study systematically investigated the dynamic changes of calcium transients in NRVMs under high-glucose (HG) induction at different time points (48, 72, 96, and 120 h). In addition to our primary findings, we observed a time-dependent characteristic: calcium transient dysfunction was prominent at 72 h but spontaneously recovered by 120 h. While this reversal likely reflects an intrinsic compensatory adaptation of NRVMs, we consider this a secondary finding that primarily serves as a useful phenomenological indicator for defining the optimal therapeutic window. Based on this, we focused on the 72 h time point (prior to overt compensation), where our results demonstrated that SMS effectively restores the Ca^2+^ handling capacity of cardiomyocytes. This finding strongly suggests that SMS can restore the Ca^2+^ handling capacity of cardiomyocytes in the early stage of high-glucose exposure, an effect consistent with results from animal experiments where SMS improved calcium transients and SR calcium transport in the early stages of DCM [14].

The normalization of calcium transients in the HG group at 120 h also provides important insights into the therapeutic window of SMS. Since calcium transient dysfunction is most obvious at 72 h (before compensatory changes), intervention at this stage may be more effective in preventing the progression of HG-induced myocardial injury, as compensatory responses often mask underlying cellular damage and may lead to long-term myocardial remodeling if not intervened in a timely manner. The ability of SMS to improve calcium transient dysfunction at 72 h suggests that it can target early HG-induced calcium dysregulation, block the progression of myocardial injury, and potentially prevent the occurrence of subsequent compensatory remodeling.

Our observation that HG induces diastolic calcium overload is consistent with previous reports linking hyperglycemia to impaired calcium homeostasis in cardiomyocytes [31,32,33]. Diastolic calcium overload is a well-established contributor to myocardial contractile dysfunction, as it disrupts the normal relaxation phase of the cardiac cycle and can trigger cellular stress pathways, including oxidative stress and apoptosis [34]. The increase in SR calcium leak observed in HG-treated cells likely plays a central role in this process, as abnormal calcium efflux from the SR would directly elevate diastolic cytosolic calcium levels and deplete SR calcium stores [35,36]. This is supported by our finding that HG reduces SR calcium content. Collectively, these factors resulted in a decreased amplitude of calcium transients and an increased decay time constant (tau) following 72 h of high-glucose induction.

The normalized SR calcium leak ratio, which accounts for differences in SR calcium content, confirmed that HG increases the relative risk of SR calcium leakage. This suggests that HG not only reduces SR calcium storage but also impairs the integrity of the SR membrane or the function of key calcium-regulating proteins, such as the RyR2 and/or SERCA2a. RyR2 hyperactivity, often driven by oxidative stress, is a major cause of SR calcium leak in pathological conditions, including diabetes and cardiac hypertrophy [37,38]. Our findings align with these established mechanisms, providing direct evidence that HG disrupts SR calcium handling in neonatal cardiomyocytes. We were more interested in identifying the mediators of this process, and thus performed pharmacological intervention assays.

Pharmacological interventions with 2%SMS-MS, NAC and KN93 effectively mitigated HG-induced calcium dysregulation. NAC, a potent antioxidant, may exert its protective effects by scavenging ROS, which are known to promote RyR2 hyperactivity and inhibit SERCA2a function. The ability of both agents to normalize SR calcium handling suggests that targeting RyR2 stability and oxidative stress are promising strategies for mitigating HG-induced myocardial injury. Moreover, KN93, an inhibitor of calcium/calmodulin-dependent protein kinase II (CaMKII), had a similar effect on calcium handling, indicating that CaMKII-dependent pathways may play a key role in this model [39,40].

As is well known, CaMKII is a multifunctional serine/threonine kinase that serves as a pivotal signaling regulator in the cardiovascular system [5] and is mainly regulated by two phosphorylation pathways: autophosphorylation, which is dependent on elevated intracellular calcium levels, and oxidative phosphorylation, which is driven by increased superoxide production. We observed an upregulation of CaMKII oxidative phosphorylation, which is likely attributable to increased ROS generation, a finding validated in our experiments. Moreover, the inhibitory effect of NAC intervention on this process confirmed that attenuating superoxide overproduction is an effective strategy to protect against high-glucose-induced myocardial calcium handling dysfunction. Concomitantly, we detected an increase in CaMKII autophosphorylation, which may result from elevated cytoplasmic calcium concentrations. The upregulation of both phosphorylation forms of CaMKII can enhance RyR activity and thereby promote calcium leakage, which also accounts for the reduced SR calcium store capacity and the subsequent attenuation of calcium transients [8,41]. Additionally, increased calcium leakage further elevates cytoplasmic calcium levels, which in turn modulates CaMKII autophosphorylation; meanwhile, the enhanced autophosphorylation and oxidative phosphorylation of CaMKII can further exacerbate calcium leakage, thus forming a vicious feedback loop [42]. Inhibition of CaMKII activity by KN93 reduced intracellular calcium concentrations and preserved the SR calcium store capacity. Collectively, increased ROS production triggers calcium overload, and the subsequent elevation of CaMKII autophosphorylation further aggravates calcium overload and calcium leakage. SMS intervention reduced ROS generation and lowered cytoplasmic calcium levels, thereby inhibiting both oxidative and autophosphorylation of CaMKII; this preservation of SR calcium store capacity also explains the attenuation of calcium transient amplitude under high-glucose conditions.

These findings have important implications for the treatment of DCM, a major complication of diabetes mellitus characterized by impaired myocardial function in the absence of coronary artery disease. Our results identify SR calcium handling dysfunction as a key early event in HG-induced cardiomyocyte injury and highlight SMS as a potential therapeutic agent for preserving calcium homeostasis and preventing myocardial dysfunction in diabetes. Previously, studies explored the effects of SMS interventions in in vivo models of type I and type II diabetes on myocytes’ calcium homeostasis, which is also related to CaMKII activity [14].

Mitochondria, the cellular power source, generate substantial ATP via oxidative phosphorylation to fuel various vital activities in cardiomyocytes, particularly the function of the SR Ca^2+^-ATPase (SERCA) during ECC [43]. High-glucose conditions can directly impair mitochondrial function, leading to electron transport chain dysfunction, reduced ATP synthesis, and excessive production of ROS [44,45]. Excessive ROS generation not only causes oxidative damage to intracellular proteins, lipids, and DNA but also exacerbates mitochondrial dysfunction, creating a vicious cycle [46]. Furthermore, there is a close crosstalk between mitochondrial function and Ca^2+^ homeostasis. On the one hand, SERCA pump activity relies on ATP supplied by mitochondria; on the other, disrupted SR Ca^2+^ homeostasis—particularly increased diastolic Ca^2+^ leak—induces cytosolic and mitochondrial matrix Ca^2+^ overload, which further compromises mitochondrial function [43].

In this study, using a high glucose in vitro model of NRVMs, we systematically evaluated the effects of SMS on mitochondrial energy metabolism, ROS generation, and calcium homeostasis, with a focus on CaMKII-related signaling. Seahorse XF analysis confirmed that while high glucose did not significantly inhibit mitochondrial basal respiration at 72 h, maximal respiration, or ATP production, the 2%SMS-MS significantly enhanced basal oxygen consumption rate (basal OCR), ATP production, and maximal respiratory capacity, while restoring the increased spare respiratory capacity induced by HG. This indicates that SMS increases ATP supply and enhances the stress resilience of mitochondria under high-glucose stress. In contrast, although the classic ROS scavenger N-acetylcysteine (NAC) and the CaMKII inhibitor KN-93 alleviated oxidative stress and CaMKII overactivation, they exhibited lower basal respiration and ATP production compared to the control under the same conditions. This suggests that single strategies of antioxidant or signal inhibition may come at the cost of partially suppressing oxidative phosphorylation, which is detrimental to overall energy supply. This contrast highlights the advantage of SMS in achieving a superior balance between inhibiting damage signals and boosting mitochondrial energy metabolic activity [47,48]. These results underscore the multi-component, multi-target, and holistic regulatory advantages of traditional Chinese medicine compound formulas. SMS is not a simple inhibitor; rather, it restores the metabolic reserve and homeostatic balance of cardiomyocytes by regulating the intracellular environment [49,50].

Consistent with the recovery of mitochondrial function, SMS significantly reduced intracellular ROS and mtROS levels induced by high glucose. After 72 h of high-glucose exposure, ROS and mtROS levels in NRVMs were significantly elevated, whereas the medicated serum and the original formula liquid were reduced both in a dose-dependent manner within a certain range; the reduction at high doses was comparable to that of the classic antioxidant NAC [51,52]. Notably, TUNEL staining revealed no significant apoptosis in cardiomyocytes at 72 h of high glucose and each drug treatment. This result suggests that within the time window of this experiment, high glucose primarily induced functional injury rather than structural death in cardiomyocytes [53]. This functional injury manifests as dysfunction of calcium-handling proteins and elevated oxidative stress levels, without initiating the apoptotic program. This indicates that the early intervention window for DCM may lie primarily in correcting cellular metabolic and functional disorders, rather than merely preventing cell death [54]. The intervention of SMS at this stage halts the progression from functional to structural damage by correcting mitochondrial function and calcium homeostasis, which holds significant clinical implications for the early prevention and treatment of DCM.

Mechanistically, Western blot results showed that high-glucose exposure for 72 h significantly upregulated Ox-CaMKII, p-CaMKII and p-RyR2 in NRVMs [55]. Intervention with 2%SMS-MS and positive controls reduced the high-glucose-induced elevation of Ox-CaMKII and p-CaMKII, and the 2%SMS-MS group also significantly inhibited the upregulation of p-RyR2. Further functional experiments indicate that by alleviating the pathological phosphorylation of RyR2, SMS reduces SR calcium leak and maintains a higher SR calcium load, thereby optimizing overall calcium transient characteristics [56]. This is highly consistent with in vivo evidence that SMS reduces SR Ca^2+^ leak and protects myocardial calcium homeostasis in diabetic mice. In summary, a potential mechanistic chain can be proposed: SMS reduces ROS generation, inhibits CaMKII oxidation and phosphorylation, and appears to decrease RyR2-mediated SR calcium leak, while simultaneously improving oxidative phosphorylation and ATP generation at the mitochondrial level [57,58]. This may achieve multi-target intervention on the pathological axis of “energy metabolism-ROS and/or calcium-CaMKII-RyR2/SR calcium homeostasis,” ultimately alleviating high-glucose-induced cardiomyocyte dysfunction. It is important to note that this proposed mechanistic pathway is supported by molecular association and pharmacological inhibition data in the present study, and lacks direct validation steps such as genetic manipulation of CaMKII or RyR2, as well as direct evidence for a causal link between SMS-mediated mitochondrial energy metabolism improvement and CaMKII inactivation. This study also has certain limitations. First, the present study is based entirely on an in vitro neonatal rat cardiomyocyte model with short-term high-glucose induction, which represents a highly simplified experimental system that cannot fully recapitulate the complex pathophysiological features of adult diabetic patients, including long-term hyperglycemia, systemic metabolic disorders such as insulin resistance and dyslipidemia, neurohumoral activation, progressive myocardial structural remodeling, and intercellular crosstalk among cardiomyocytes, fibroblasts, and endothelial cells. Neonatal cardiomyocytes exhibit an immature phenotype distinct from adult cardiomyocytes, with retained proliferative capacity, a metabolic preference for glycolysis over fatty acid oxidation, different isoform expression of calcium-handling proteins, and incomplete mitochondrial maturation. These characteristics may endow neonatal cardiomyocytes with greater metabolic flexibility and higher tolerance to hyperglycemic stress relative to terminally differentiated adult cardiomyocytes. Accordingly, the compensatory mechanisms observed in the present study, such as the upregulation of spare respiratory capacity, may not fully represent the chronic pathological adaptations that occur in adult diabetic cardiomyopathy. In particular, the recovery of calcium transients observed at 120 h must be interpreted with caution, as the current data cannot definitively distinguish between intrinsic metabolic adaptation, selective survival of a resilient subpopulation of cardiomyocytes, or culture-related artifacts/phenotypic drift over the extended time course. The underlying mechanism of this time-dependent recovery remains to be fully elucidated and will be the subject of our future investigations. Second, this study mainly explored the protective mechanism of SMS from the perspectives of CaMKII/RyR2-mediated calcium homeostasis and mitochondrial energy metabolism, without systematically investigating other critical metabolic and antioxidant pathways, including AMPK, SIRT, and Nrf2, nor dissecting the specific contribution of each monomer component of SMS. Third, the proposed mechanistic link between CaMKII/RyR2 signaling and SMS-regulated calcium homeostasis was supported by pharmacological inhibition (e.g., KN93) and correlative molecular evidence, but lacks direct genetic validation, such as gene knockout or site-directed mutagenesis, to establish a strict causal relationship. Similarly, direct experimental evidence supporting the upstream regulatory effect of SMS-improved mitochondrial function on CaMKII activity is still insufficient and needs to be further verified in future investigations. In addition, it should be acknowledged that SMS is a multi-component natural product with a complex chemical composition. Although the multi-component nature may simultaneously target multiple pathological pathways and thus represent a potential advantage in the treatment of diabetic cardiomyopathy, this complexity also brings inherent challenges, including potential batch-to-batch variability in component contents and proportions. In the present study, the protective effects of SMS were evaluated at the crude extract level, and the precise bioactive components responsible for the observed effects on CaMKII/RyR2-mediated calcium homeostasis and mitochondrial function remain to be identified. Therefore, further studies focusing on component isolation, identification, and individual validation are warranted to clarify the key active monomers, their respective contributions, and potential synergistic mechanisms, which will help improve the stability, controllability, and translational potential of SMS-based interventions.

## 4. Materials and Methods

### 4.1. Isolation, Culture, and Grouping of Neonatal Rat Cardiomyocytes

This study strictly followed the principles of the “Guide for the Care and Use of Laboratory Animals” published by the US National Institutes of Health (NIH Publication No. 85-23, revised 1996). All experimental protocols and operations strictly adhered to the ethical guidelines for the use of laboratory animals and were approved by the Animal Ethics Committee of the Experimental Center, China Academy of Chinese Medical Sciences (Approval Number: [ERCCACMS21-2106-14]). The 1-day-old Sprague-Dawley rats were obtained from Beijing Vital River Laboratory Animal Technology (Beijing, China). Primary NRVMs were isolated from 1-day-old Sprague-Dawley rats following a previously reported protocol with modifications [59]. In brief, the rats were immersed in 75% ethanol for a few seconds, after which the thoracic cavity was rapidly incised along the left midclavicular line with ophthalmic scissors in the right hand to harvest the hearts. The isolated hearts were rinsed three times in pre-chilled PBS at 4 °C; the atria and large blood vessels were then removed, and the ventricular myocardium was dissected and cut into 1 mm^3^ tissue pieces. Myocardial dissociation was performed using the Miltenyi Biotec Neonatal Heart Dissociation Kit with a gentle MACS tissue processor. The dissociated cell suspension was seeded into culture flasks and subjected to differential adhesion for 40 min in a 37 °C incubator with 5% CO_2_ to remove fibroblasts. Subsequently, the non-adherent cell suspension was transferred to new culture flasks, and 5-bromo-2′-deoxyuridine (BRDU) was added at a final concentration of 0.1 mmol·L^−1^ to inhibit residual fibroblast proliferation. Isolated NRVMs were seeded and maintained in low-glucose DMEM (5.5 mM glucose) supplemented with 10% FBS and 100 U/mL penicillin–streptomycin, and cultured for 24 h prior to grouping for subsequent experiments.

In the dynamic changes of calcium transient assay at different time points (48, 72, 96, and 120 h), the cell groups were divided into two groups: (1) The LG group—low-glucose (5.5 mM) + mannitol (27.8 mM) DMEM; (2)The HG group (HG)—high-glucose (33.3 mM) DMEM.

The cell groups were as follows. (1) Control group (LG): low-glucose (5.5 mM) + mannitol (27.8 mM) DMEM; (2) model group (HG): high-glucose (33.3 mM) DMEM; (3) HG + 2% SMS serum group (2%SMS-MS): HG DMEM + 2%SMS-MS; (4) HG + 5% SMS serum group (5%SMS-MS): HG DMEM + 5%SMS-MS; (5) HG + 10% SMS serum group (10%SMS-MS): HG DMEM + 10%SMS-MS; (6) HG + 20% SMS serum group (20%SMS-MS): HG DMEM + 20%SMS-MS; (7) NAC group (NAC): HG DMEM + 1 mM NAC (ROS scavenger); and (8) KN93 group (KN93): HG DMEM + 10 µM KN93 (CaMKII inhibitor). In all groups, without the addition of drug-containing serum, a 5% control group serum was added. In this study, NAC and KN-93 were set as positive control groups for the pharmacological treatment of DCM. To exclude the potential confounding effects of hyperosmolarity, an osmotic control was established. Mannitol was added to the LG group at a concentration of 27.8 mM (LG: 5.5 mM glucose + 27.8 mM mannitol) to strictly match the osmolarity of the HG group (33.3 mM glucose).

### 4.2. Preparation of SMS-MS

Specific-pathogen-free (SPF) adult male Sprague-Dawley (SD) rats, weighing approximately 300 g, were obtained from Beijing Vital River Laboratory Animal Technology Co., Ltd. (Beijing, China). These animals were randomly assigned to either a blank control group or a SMS group (*n* = 6 per group). To prepare the SMS aqueous extract, *Panax ginseng*, *Ophiopogon japonicus*, and *Schisandra chinensis* were mixed at a crude drug ratio of 1:2:1, with the final concentration standardized to contain 0.1 g of *P. ginseng* crude drug per milliliter. Rats in the SMS group were intragastrically administered the extract at a dose of 1050 mg/kg/day (equivalent to the clinical adult daily dose based on body surface area conversion), divided into two equal doses (morning and evening) for three consecutive days. An equivalent volume of double-distilled water was given to the blank control group under identical conditions. Two hours after the final administration, blood samples were collected from the abdominal aorta under anesthesia for serum preparation. Blood samples were collected from SD rats, and serum was isolated by centrifugation. To minimize batch-to-batch variation and ensure consistency, serum added to cultured cells in each group was prepared as a pooled mixture from 10 rats within the same group. All serum samples were filtered, aliquoted, and stored under identical conditions to avoid experimental variability.

### 4.3. Detection of Apoptosis in Neonatal Rat Cardiomyocytes

The effect of SMS on high-glucose-induced apoptosis in NRVMs was detected using a TUNEL cell apoptosis in situ detection kit (Beyotime; Shanghai, China). Cells in each group were rinsed with PBS twice for 3 min each, then fixed with −20 °C methanol for 15 min. After rinsing with PBS three times for 3 min each, 50 µL of TUNEL reaction solution was added and incubated at 37 °C in the dark for 1 h. Cells were rinsed with PBS twice for 3 min each, and then stained with Hoechst 33258 (Beyotime; Shanghai, China) dye at 37 °C in the dark for 10 min. Nuclear morphology was observed using a fluorescence microscope. Six high-power fields were randomly selected from each image, and the apoptotic index (AI) was calculated as follows: AI (%) = (number of TUNEL-positive cells/total number of cells) × 100%.

### 4.4. Detection of Calcium Transients and SR Calcium Homeostasis in Neonatal Rat Cardiomyocytes

#### 4.4.1. Assessment of Ca^2+^ Transients and SR Calcium Leak in Cultured NRVMs

NRVMs cultured in 96-well plates were loaded with 2 μM Fura-4 AM (Invitrogen, Carlsbad, CA, USA) in Tyrode’s solution for 30 min at 35 °C in the dark. Post-loading, the cells were washed twice with Tyrode’s buffer to eliminate unbound dye. A pair of platinum wire electrodes was inserted into each well, and the cardiomyocytes were electrically stimulated at 1 Hz (4 ms pulse duration) at room temperature. The cells were then exposed to excitation light at 460–490 nm, and the emitted fluorescence signal was detected at 515–555 nm. Calcium fluorescence in the cardiomyocytes was recorded using an Andor EMCCD camera (Belfast, UK) at an acquisition frequency of ~120 Hz.

For the SR calcium leak assay, the following protocol was performed. Following 60 s of Ca^2+^ transient induction at 1 Hz in Tyrode’s solution, the perfusate was switched to Na^+^/Ca^2+^-free solution (containing 140 mM LiCl, 5.4 mM KCl, 1 mM MgCl_2_, 1 mM EGTA, 10 mM HEPES, and 10 mM D-glucose, adjusted to pH 7.4 with LiOH) supplemented with 1 mM tetracaine (TET, 580742, J&K Scientific, Beijing, China). This treatment reduced intracellular Ca^2+^ levels and blocked Ca^2+^ influx/efflux through the plasma membrane and RyRs. After 40 s of TET perfusion, the cells were washed with 0Ca0Na solution to restore RyR-mediated Ca^2+^ leak from the SR. SR Ca^2+^ leak was calculated based on the change in cytosolic Ca^2+^ fluorescence intensity using the formula [(F_0Ca0Na_ − F_tet_)/F_tet_], where F_0Ca0Na_ represents the fluorescence intensity in 0Ca0Na solution, and F_tet_ represents the fluorescence intensity in the presence of TET.

To measure total SR Ca^2+^ content, a 20 mM caffeine solution was rapidly perfused onto the cells using a 250 μM diameter perfusion tip nearby the cells to trigger complete SR Ca^2+^ release. The amplitude of the Ca^2+^ transient induced by caffeine (F_caff_) was used to quantify SR Ca^2+^ load, calculated as [(F_caff_ − F_tet_)/F_tet_]. A normalized SR Ca^2+^ leak index was determined using the formula [(F_0Ca0Na_ − F_tet_)/(F_caff_ − F_tet_)/F_tet_] [60], which accounts for differences in total SR Ca^2+^ content.

#### 4.4.2. Measurement of Intracellular Free Ca^2+^ in Cultured NRVMs

Cultured neonatal cardiomyocytes were loaded with 2 μM Fura-2 AM (Invitrogen, Carlsbad, CA, USA) in Tyrode’s solution (composed of 137 mM NaCl, 5.4 mM KCl, 1.2 mM MgCl_2_, 10 mM HEPES, 10 mM D-glucose, 1.2 mM NaH_2_PO_4_, and 1.8 mM CaCl_2_, pH 7.4) for 30 min at 35 °C in the dark. After the loading period, the cells were rinsed twice with Tyrode’s solution to remove excess Fura-2 AM. Intracellular free Ca^2+^ levels were detected using an Andor iXon3 EMCCD camera (Andor, Belfast, UK) attached to a 10× fluorescence objective. Excitation light (340 ± 10 nm and 380 ± 10 nm) was supplied by a Sutter DG4 xenon lamp system (Sutter, Sacramento, CA, USA), and the emitted fluorescence was measured at 510 ± 25 nm. The use of ratiometric fluorescence requires switching between two excitation wavelengths, resulting in a relatively low sampling frequency of approximately 10 Hz. Despite the low sampling rate, there was still a relatively stable calcium signal during diastole, which we used as the diastolic calcium level. The diastolic intracellular Ca^2+^ concentration was expressed as the ratio of fluorescence intensities at 340 nm and 380 nm excitation wavelengths (F_340_/F_380_).

### 4.5. Mitochondrial Stress Test in Cardiomyocytes

Grouped cells were cultured in XF-specific culture plates. Cellular OCR was quantitatively measured using a Bioscience Seahorse XFe Analyzer and the Mito Stress Test kit (Agilent; Santa Clara, CA, USA) to obtain parameters such as basal respiration rate, ATP production, proton leak, and maximal respiration rate. The determination of cellular OCR was as follows. ① Basal oxygen consumption: Includes oxygen consumption from mitochondrial oxidative phosphorylation and proton leak. That is, after protons form potential energy through the respiratory chain on the mitochondrial membrane, a portion of the protons flows back through ATP synthase to form ATP. Another portion passes through the mitochondrial membrane but only undergoes oxidation, converting potential energy into heat. ② ATP production: The decrease in oxygen consumption after adding the ATP synthase inhibitor oligomycin represents the oxygen consumption used by the body for ATP synthesis, indirectly indicating the ATP production of the cells at that time. ③ Maximal oxygen consumption capacity: The uncoupler FCCP is added; as a proton carrier, it causes a large backflow of protons and massive oxygen consumption, but this proton backflow cannot form ATP. The increase in oxygen consumption after FCCP represents the maximal oxygen consumption capacity of the mitochondria, indirectly indicating maximal respiratory capacity. ④ Maximal respiration rate: Finally, the respiratory chain inhibitors antimycin A and oligomycin are added to completely stop mitochondrial oxygen consumption; the difference between this value and the highest point represents the maximal respiration rate. OCR measurements were normalized to cell number. Specifically, approximately 4000 cells were seeded into each well of the Seahorse 96-well plate (Agilent, Santa Clara, CA, USA) to ensure consistent cell density across all groups.

### 4.6. Detection of Intracellular ROS and Mitochondrial ROS

Grouped cells were cultured in glass-bottom dishes and loaded with the probe in situ: DCFH-DA (Beyotime, Shanghai, China) was diluted with Tyrode’s solution at a ratio of 1:1000 to a final concentration of 10 µmol/L. Cells were incubated at room temperature for 30 min. Cells were washed three times with Tyrode’s solution to fully remove DCFH-DA that had not entered the cells. For mitochondrial ROS (mtROS) detection, cells were stained at 37 °C for 30 min with 2 μmol/L MitoSOX Red (Thermo Fisher Scientific, Waltham, MA, USA) in Tyrode’s solution. After three Tyrode’s solution washes to eliminate background fluorescence, both intracellular ROS and mtROS levels were detected using the Echo Revolve Intelligent Microscope Imaging System (Echo Laboratories, San Diego, CA, USA).

### 4.7. Western Blot

Based on the molecular weight of the target proteins, the concentration of the stacking gel was 5%. Western blot detection was performed for relevant proteins: the protein expression levels of p-CaMKII, Ox-CaMKII, total-CaMKII, total-RyR2 and p-RyR2. Band intensities were quantified using ImageJ software (Version 2.14.0). Changes in protein content were determined by calculating the ratio to the GAPDH internal reference. Antibody dilution: CaMKII Rabbit Monoclonal Antibody (1:1000); Phospho-CaMKII (Thr286) (D21E4) Rabbit Monoclonal Antibody (1:1000); Anti-oxidized-CaM Kinase II (Met281/282) Antibody (1:1000); Anti-CaMKII Antibody [EP1829Y] (1:1000); Anti-Ryanodine receptor 2/RYR-2 Antibody [EPR26288-70] (1:1000); Ryanodine Receptor 2 (RYR2) (pSER2814) pAB (1:2000); GAPDH (D16H11) Rabbit Monoclonal Antibody (1:1000); and Multi-rAb™ HRPGoat Anti-Rabbit Recombinant Secondary Antibody (1:5000).

### 4.8. Determination of Active Components of SMS-MS

SMS consists of Ginseng, Schisandra, and Ophiopogon. Precisely aspirate 200 µL of the SMS-MS sample into a 1.5 mL centrifuge tube, add 600 µL of methanol, and ultrasonically extract the mixture for 30 min. Subsequently, centrifuge at 4 °C and 12,000 rpm for 10 min. Precisely aspirate 100 µL of the supernatant and transfer it to a sample vial for UHPLC-Q-Orbitrap HRMS analysis. Chromatographic separation was performed using an ACQUITY UPLC HSS T3 column (2.1 mm × 100 mm, 1.8 µm, Atlanta, GA, USA). Mobile phase A was water (containing 0.1% formic acid), and mobile phase B was acetonitrile. The gradient elution program was as follows: 0–1.0 min, 98% A; 1.0–14.0 min, 98% A linearly changed to 70% A; 14.0–25.0 min, 70% A linearly changed to 0% A; 25.0–28.0 min, 0% A; and 28.1–30.0 min, 98% A for equilibration. The flow rate was 0.3 mL/min, the column temperature was 40 °C, and the injection volume was 6.0 µL. Mass spectrometry analysis was performed using a Q-Exactive high-resolution mass spectrometer (Q Exactive Thermo Fisher Scientific, Waltham, MA, USA) equipped with a heated electrospray ionization source (HESI), acquiring data in positive and negative ion modes. Ion source parameters were set as follows: ion spray voltages were +3.7 kV (positive mode) and −3.5 kV (negative mode); capillary temperature was 320 °C; auxiliary gas heating temperature was 300 °C; sheath gas pressure was 30 psi; auxiliary gas pressure was 10 psi; and collision gas pressure was 1.5 mTorr. Sheath gas, auxiliary gas, and collision gas were all nitrogen. Data acquisition mode was full scan/dd-MS2. Full scan parameters: resolution was 70,000; automatic gain control (AGC) target was 1 × 10^6^; maximum injection time was 50 ms; mass-to-charge ratio scan range was 100–1500. dd-MS2 parameters: resolution was 17,500; AGC target was 1 × 10^5^; maximum injection time was 50 ms; mass isolation window was *m*/*z*^2^; collision energies were 10 V, 30 V, and 60 V; and intensity threshold was 1 × 10^5^. Raw data processing was performed using Progenesis QI 3.0 software (Waters, Milford, MA, USA), including raw data import, peak extraction, and adduct deconvolution. Mass spectrometry data were acquired in full scan/dd-MS2 mode. The full scan parameters were set as follows: resolution of 70,000, automatic gain control (AGC) target of 1 × 10^6^, maximum injection time of 50 ms, and scan range *m*/*z* of 100–1500. The dd-MS2 parameters were: resolution 17,500, AGC target 1 × 10^5^, maximum injection time 50 ms, isolation window *m*/*z*^2^, and stepped collision energies of 10, 30, and 60 V. Components in SMS-MS were conducted by searching against a reference database (TCM Pro 2.0, Beijing Hexin Technology Co., Ltd., Beijing, China) and a theoretical database (constructed based on the literature and public databases), combining multi-dimensional information such as a reference retention time error of <0.5 min, a parent ion mass error and fragment mass error of <5 ppm, an MS2 fragment matching degree of >60, and an isotopic distribution of >80%.

### 4.9. Methods of Network Pharmacology Analysis

#### 4.9.1. Prediction of SMS Serum Compound Targets

Rat serum samples were collected after SMS administration, and eight compounds were identified: Ginsenoside Rb1, Ginsenoside Rd, Schisandrol A, Gomisin D, Ginsenoside Rg3, Ginsenoside Rg5, Schisanhenol, and Schisandrin B. By reviewing previous studies in PubMed (https://pubmed.ncbi.nlm.nih.gov/, accessed on 17 March 2026), the Canonical SMILES and INCHI numbers of these active compounds were retrieved from the PubChem database (https://pubchem.ncbi.nlm.nih.gov/, accessed on 17 March 2026). These were then input into SwissTarget Prediction (http://www.swisstargetprediction.ch/, accessed on 17 March 2026), BATMAN (http://bionet.ncpsb.org.cn/, accessed on 17 March 2026), and TCMSP databases (https://www.tcmsp-e.com/load_intro.php?id=43, accessed on 17 March 2026) to obtain predicted targets for the above compounds.

#### 4.9.2. Collection of Disease Targets and SMS-Disease Intersection Targets

“Diabetic cardiomyopathy,” “myocardial dysfunction,” “systolic dysfunction,” and “diastolic dysfunction” were used as keywords to search the GeneCards (https://www.genecards.org/, accessed on 17 March 2026) and OMIM databases (https://www.omim.org/, accessed on 17 March 2026). The UniProt database (https://www.uniprot.org/, accessed on 17 March 2026) “Retrieve/ID Mapping” was used to standardize disease targets. Venny 2.1 (https://bioinfogp.cnb.csic.es/tools/venny/, accessed on 17 March 2026) was used to obtain the intersection of SMS-disease targets.

#### 4.9.3. Protein–Protein Interaction (PPI) Network Construction and Core Target Analysis

The STRING database (https://cn.string-db.org/, accessed on 18 March 2026) and Cytoscape 3.7.1 were used to screen core targets and construct the PPI network.

#### 4.9.4. Enrichment Analysis

To further explore the potential biological mechanisms by which Shengmai San (SMS) ameliorates calcium homeostasis imbalance in cardiomyocytes, this study used the DAVID database (https://davidbioinformatics.nih.gov/) to perform GO (Gene Ontology) and KEGG (Kyoto Encyclopedia of Genes and Genomes) enrichment analysis on the screened core targets, with *p* < 0.05 and species set as Homo sapiens. GO analysis included biological process (BP), cellular component (CC), and molecular function (MF).

### 4.10. Statistical Methods

Data are presented as mean ± standard deviation (SD). Statistical comparisons were performed using GraphPad Prism software (Version 10.0.0). For comparisons among three or more groups, one-way analysis of variance (ANOVA) was used, followed by Tukey’s post hoc test for multiple comparisons. Tukey’s test automatically corrects for multiple comparisons to control the type I error rate. A value of *p* < 0.05 was considered statistically significant, and *p* < 0.01 was considered extremely significant. Data for each group were pooled from 6 independent biological replicates, consisting of cardiomyocytes isolated from 24 h old neonatal SD rats (10 pups per batch). For single-cell measurements, each data point represents one individual cell. For Seahorse extracellular flux analysis, data were derived from 6 independent cell isolations, with *n* = 45 technical replicates per group. For Western blot analysis, results were obtained from 3 independent cell isolations (10 pups per batch), with three data points representing three independent biological replicates.

### 4.11. Drugs and Reagents

SMS solution (medicinal decoction formulated on the basis of Shengmai San) is a listed Chinese medicine preparation (OTC Z22022398) from Jilin Tianqiang Pharmaceutical Co. (Tonghua, China). Miltenyi Biotec Neonatal Heart Dissociation Kit (Cat# 130-098-373) was obtained from Miltenyi Biotec GmbH (DE, Bergisch Gladbach, Germany). The 5-bromo-2′-deoxyuridine (BRDU) (Cat# HY-15910) was acquired from MedChemExpress (MCE, Monmouth Junction, NJ, USA). The following reagents were supplied by Thermo Fisher Scientific (Waltham, MA, USA): DMEM (Cat# 11965092), Mitosox Red (Cat# M36008), penicillin–streptomycin (Cat# 15140122), FBS (Cat# A5256701), Fura-4 AM (Cat# M14206) and Fura-2 AM (Cat# F1221). The following assay kits and reagents were sourced from Beyotime Biotechnology (Shanghai, China): Hoechst 33342 (Cat# C1026),KN-93 Phosphate (Cat# SD9536), NAC (ROS scavenger) (Cat# ST1546), Colorimetric TUNEL Apoptosis Assay Kit (Cat# C1091) and Reactive Oxygen Species Assay Kit with DCFH-DA (Cat# S1105S). Primary antibodies included: Phospho-CaMKII (Thr286) (D21E4) Rabbit Monoclonal Antibody (Cat# 12716) and GAPDH (D16H11) Rabbit Monoclonal Antibody (Cat# #5174) from Cell Signaling Technology (Danvers, MA, USA), Anti-oxidized-CaM Kinase II (Met281/282) Antibody (Cat# 07-1387) from Merck KGaA (Darmstadt, Germany), Multi-rAb™ HRPGoat Anti-Rabbit Recombinant Secondary Antibody (H+L, Cat# RGAR001) from Proteintech Group, Inc. (Wuhan, China). Tetracaine (TET, Cat #580742) was supplied by J&K Scientific Ltd. (Beijing, China). Seahorse XF Media, Supplements & Calibrant (Cat# 103680-100), XFe96 Spheroid Microplates (Cat# 102978-100), and Seahorse XF Cell Mito Stress Test Kit (Cat# 103010-100) were purchased from Agilent Technologies Inc. (Santa Clara, CA, USA).

## 5. Conclusions

In conclusion, the present study extends these findings by focusing specifically on early hyperglycemic injury in vitro, providing novel mechanistic insights into oxidative CaMKII activation, sarcoplasmic reticulum Ca^2+^ leak, and the crosstalk between calcium handling and mitochondrial dysfunction at the initial stage of high-glucose exposure. Our results demonstrate that SMS effectively improves high-glucose-induced mitochondrial dysfunction and calcium homeostasis imbalance in neonatal rat cardiomyocytes. Our results suggest that SMS modulates the ROS–CaMKII–calcium axis during the early stages of hyperglycemic injury. This study improves our understanding of the early pathological mechanisms underlying diabetic cardiomyocyte damage and highlights SMS as a promising candidate for further mechanistic exploration and translational research. This finding provides new experimental evidence and theoretical support for the prevention and treatment of DCM.

## Data Availability

The original contributions presented in this study are included in the article/Supplementary Materials. Further inquiries can be directed to the corresponding author.

## Supplementary Materials

The following supporting information can be downloaded at: https://www.mdpi.com/article/10.3390/ph19040601/s1, Table S1. Identification of the prototype compounds of Shengmai San in rats serum. Table S2. GO_BP enrichment analysis results. Table S3. GO_CC enrichment analysis results. Table S4. GO_MF enrichment analysis results. Table S5. KEGGt analysis results.
