# Supplementary material for: Shengmai San Ameliorates High-Glucose-Induced Calcium Homeostasis Imbalance via Improving Energy Metabolism in Neonatal Rat Cardiomyocytes
Source: Pharmaceuticals (Basel). 2026 Apr 8;19(4):601. doi: 10.3390/ph19040601 (PMC13119379; doi:10.3390/ph19040601)
Supplement: Supplementary file 1 [file pharmaceuticals-19-00601-s001.zip › pharmaceuticals-4168064-supplementary.pdf]

Table S1. Identification of the prototype compounds of Shengmai San in rats serum.

NO.	**tR/ min**	**Identification**	**Experimental m/z**	Theoretical m/z	Peak area	Formula
1	17.72	Ginsenoside Rb1	1109.613	1109.6102	8840	$C_{54}H_{92}O_{23}$
2	18.53	Ginsenoside Rd	991.5471	991.5483	1394	$C_{48}H_{82}O_{18}$
3	20.02	Schisandrol A	433.2215	433.2221	10443	$C_{24}H_{32}O_{7}$
4	20.26	Gomisin D	531.2217	531.2225	417728	$C_{28}H_{34}O_{10}$
5	20.47	Ginsenoside Rg3	829.4988	829.4955	3149	$C_{42}H_{72}O_{13}$
6	22.07	Ginsenoside Rg5	767.4924	767.494	6275	$C_{42}H_{70}O_{12}$
7	22.38	Schisanhenol	403.2105	403.2115	3619	$C_{23}H_{30}O_{6}$
8	23.84	Schisandrin B	401.1952	401.1959	6832	$C_{23}H_{28}O_{6}$

Table S2. GO_BP enrichment analysis results

**Category**	**Term**	**Count**	**P-Value**	**User Ids**
GOTERM_BP	cellular response to vascular endothelial growth factor stimulus	4	0.00000295	FLT1,AKT1,MAPK14,VEGFA
GOTERM_BP	positive regulation of MAPK cascade	5	0.0000358	AR,FLT1,ADRB2,RAF1,VEGFA
GOTERM_BP	positive regulation of gene expression	6	0.0000598	AR,NOS3,AKT1,PSEN1,MAPK14,VEGFA
GOTERM_BP	intracellular calcium ion homeostasis	4	0.000128	RYR2,PLN,PSEN1,DRD3
GOTERM_BP	response to muscle stretch	3	0.000135	RYR2,RAF1,MAPK14
GOTERM_BP	regulation of cardiac muscle contraction by regulation of the release of sequestered calcium ion	3	0.000248	RYR2,PLN,CACNA1C
GOTERM_BP	MAPK cascade	4	0.000253	AR,ABCC9,RAF1,MAPK14
GOTERM_BP	negative regulation of blood pressure	3	0.000286	NOS3,ABCC9,DRD3
GOTERM_BP	calcium ion transport	4	0.000326	RYR2,PLN,NOS3,CACNA1C
GOTERM_BP	vascular endothelial growth factor receptor signaling pathway	3	0.000327	FLT1,MAPK14,VEGFA
GOTERM_BP	insulin-like growth factor receptor signaling pathway	3	0.000371	AR,AKT1,RAF1

GOTERM_BP	blood vessel diameter maintenance	3	0.000467	NOS3,ABCC9,ADRB2
GOTERM_BP	response to hypoxia	4	0.000485	RYR2,PSEN2,ABCC9,VEGFA
GOTERM_BP	lipopolysaccharide-mediated signaling pathway	3	0.000493	NOS3,AKT1,MAPK14
GOTERM_BP	blood circulation	3	0.000574	PLN,NOS3,ABCC9
GOTERM_BP	positive regulation of blood vessel endothelial cell migration	3	0.000854	NOS3,AKT1,VEGFA
GOTERM_BP	vasodilation	3	0.000996	NOS3,ABCC9,VEGFA
GOTERM_BP	negative regulation of apoptotic process	5	0.00106	AKT1,ABCC9,PSEN1,RAF1,VEGFA
GOTERM_BP	blood vessel development	3	0.00171	ABCC9,PSEN1,VEGFA
GOTERM_BP	gene expression	4	0.00175	AR,AKT1,ABCC9,PSEN1
GOTERM_BP	epithelial cell proliferation	3	0.00176	AR,PSEN1,VEGFA
GOTERM_BP	angiogenesis	4	0.00193	FLT1,NOS3,MAPK14,VEGFA
GOTERM_BP	positive regulation of transcription by RNA polymerase II	6	0.00338	AR,AKT1,ADRB2,RAF1,MAPK14,VEGFA
GOTERM_BP	apoptotic process	5	0.00435	AKT1,PSEN1,RAF1,MAPK14,VEGFA
GOTERM_BP	Notch signaling pathway	3	0.0048	PLN,PSEN2,PSEN1
GOTERM_BP	protein maturation	3	0.00639	AKT1,PSEN1,MAPK14
GOTERM_BP	response to fluid shear stress	2	0.00695	NOS3,AKT1
GOTERM_BP	calcium ion transmembrane transport	3	0.0074	RYR2,CACNA1C,PSEN1
GOTERM_BP	Notch receptor processing	2	0.00782	PSEN2,PSEN1
GOTERM_BP	positive regulation of angiogenesis	3	0.00838	FLT1,NOS3,VEGFA
GOTERM_BP	negative regulation of calcium ion transport	2	0.00868	PLN,NOS3
GOTERM_BP	amyloid-beta formation	2	0.00868	PSEN2,PSEN1
GOTERM_BP	intracellular signal transduction	4	0.00875	PSEN2,AKT1,PSEN1,MAPK14
GOTERM_BP	regulation of ventricular cardiac muscle cell action potential	2	0.00954	RYR2,CACNA1C
GOTERM_BP	positive regulation of cell population proliferation	4	0.0108	AR,FLT1,ILK,VEGFA
GOTERM_BP	cell communication by electrical coupling involved in cardiac conduction	2	0.0113	RYR2,CACNA1C
GOTERM_BP	calcium ion transmembrane import into cytosol	2	0.0113	RYR2,CACNA1C

GOTERM_BP	cardiac conduction	2	0.0113	ABCC9,CACNA1C
GOTERM_BP	nitric oxide metabolic process	2	0.0113	NOS3,AKT1
GOTERM_BP	positive regulation of intracellular estrogen receptor signaling pathway	2	0.0121	AR,HDAC6
GOTERM_BP	amyloid precursor protein catabolic process	2	0.0121	PSEN2,PSEN1
GOTERM_BP	calcium ion transport into cytosol	2	0.0121	RYR2,CACNA1C
GOTERM_BP	cell differentiation	5	0.013	FLT1,ILK,AKT1,RAF1,VEGFA
GOTERM_BP	cellular response to calcium ion	2	0.0147	AKT1,ABCC9
GOTERM_BP	negative regulation of G protein-coupled receptor signaling pathway	2	0.0147	ADRB2,DRD3
GOTERM_BP	regulation of sodium ion transport	2	0.0164	NOS3,ADRB2
GOTERM_BP	striated muscle cell differentiation	2	0.0173	AKT1,MAPK14
GOTERM_BP	positive regulation of peptidyl-serine phosphorylation	2	0.0173	AKT1,RAF1
GOTERM_BP	fibroblast migration	2	0.0173	ILK,AKT1
GOTERM_BP	vascular endothelial growth factor signaling pathway	2	0.0173	FLT1,VEGFA
GOTERM_BP	fatty acid oxidation	2	0.0181	ABCC9,MAPK14
GOTERM_BP	Schwann cell development	2	0.0181	ILK,RAF1
GOTERM_BP	membrane protein ectodomain proteolysis	2	0.0207	PSEN2,PSEN1
GOTERM_BP	nitric oxide biosynthetic process	2	0.0207	NOS3,AKT1
GOTERM_BP	protein phosphorylation	3	0.0212	AKT1,TNNI3K,RAF1
GOTERM_BP	in utero embryonic development	3	0.0214	AR,NOS3,VEGFA
GOTERM_BP	regulation of cardiac muscle contraction	2	0.0216	RYR2,TNNI3K
GOTERM_BP	cell population proliferation	3	0.0222	ILK,AKT1,VEGFA
GOTERM_BP	transcription by RNA polymerase II	3	0.0223	AR,ADRB2,MAPK14
GOTERM_BP	cell migration involved in sprouting angiogenesis	2	0.0224	AKT1,VEGFA
GOTERM_BP	negative regulation of proteolysis	2	0.0233	AKT1,HDAC6
GOTERM_BP	membraneless organelle assembly	2	0.0241	AR,HDAC6
GOTERM_BP	heart development	3	0.0242	ABCC9,CACNA1C,PSEN1

GOTERM_BP	negative regulation of extrinsic apoptotic signaling pathway via death domain receptors	2	0.0258	NOS3,RAF1
GOTERM_BP	positive regulation of cell migration	3	0.0275	FLT1,AKT1,VEGFA
GOTERM_BP	cell migration	3	0.0285	FLT1,AKT1,VEGFA
GOTERM_BP	blood vessel morphogenesis	2	0.0292	FLT1,VEGFA
GOTERM_BP	sprouting angiogenesis	2	0.0309	FLT1,VEGFA
GOTERM_BP	response to testosterone	2	0.0309	AR,PLN
GOTERM_BP	regulation of postsynapse organization	2	0.0309	AKT1,PSEN1
GOTERM_BP	negative regulation of protein-containing complex assembly	2	0.0318	RAF1,HDAC6
GOTERM_BP	positive regulation of D-glucose import	2	0.0326	AKT1,MAPK14
GOTERM_BP	regulation of heart contraction	2	0.0326	PLN,CACNA1C
GOTERM_BP	signal transduction	6	0.0333	AR,AKT1,ADRB2,RAF1,MAPK14,DRD3
GOTERM_BP	regulation of cytosolic calcium ion concentration	2	0.0335	RYR2,PLN
GOTERM_BP	homeostasis of number of cells within a tissue	2	0.0335	NOS3,VEGFA
GOTERM_BP	response to heat	2	0.036	NOS3,AKT1
GOTERM_BP	calcium ion homeostasis	2	0.036	PSEN2,PSEN1
GOTERM_BP	positive regulation of protein import into nucleus	2	0.036	PSEN1,MAPK14
GOTERM_BP	negative regulation of gene expression	3	0.0367	AKT1,PSEN1,VEGFA
GOTERM_BP	regulation of neuron projection development	2	0.0377	AKT1,PSEN1
GOTERM_BP	regulation of heart rate	2	0.0385	RYR2,TNNI3K
GOTERM_BP	endothelial cell migration	2	0.0402	NOS3,VEGFA
GOTERM_BP	thymus development	2	0.0435	PSEN1,RAF1
GOTERM_BP	cellular response to amyloid-beta	2	0.0435	ADRB2,PSEN1
GOTERM_BP	cellular response to stress	2	0.0452	AKT1,MAPK14
GOTERM_BP	visual learning	2	0.0469	PLN,DRD3
GOTERM_BP	response to hormone	2	0.0494	NOS3,AKT1

Table S3. GO_CC enrichment analysis results

Category	Term	Count	P-Value	User Ids
GOTERM_CC	protein-containing complex	8	0.00000164	RYR2,AR,PLN,PSEN2,AKT1,ABCC9,PSEN1,HDAC6
GOTERM_CC	sarcolemma	4	0.000114	RYR2,ABCC9,CACNA1C,PSEN1
GOTERM_CC	plasma membrane	13	0.000385	RYR2,FLT1,NOS3,PSEN2,ILK,ABCC9,CACNA1C,ADRB2,PSEN1,AR,AKT1,RAF1,DRD3
GOTERM_CC	sarcomere	3	0.00119	RYR2,ILK,ABCC9
GOTERM_CC	membrane	15	0.00122	RYR2,FLT1,NOS3,PSEN2,ILK,ABCC9,CACNA1C,ADRB2,PSEN1,VEGFA,AR,PLN,AKT1, RAF1,DRD3
GOTERM_CC	cytoplasm	13	0.00544	FLT1,NOS3,ILK,ABCC9,CACNA1C,TNNI3K,PSEN1,MAPK14,HDAC6,VEGFA,AR,AKT1, RAF1
GOTERM_CC	Golgi apparatus	6	0.00562	NOS3,PSEN2,ADRB2,PSEN1,RAF1,VEGFA
GOTERM_CC	gamma-secretase complex	2	0.00571	PSEN2,PSEN1
GOTERM_CC	cell cortex	3	0.0101	ILK,AKT1,PSEN1
GOTERM_CC	mitochondrion	6	0.0132	PLN,AKT1,ABCC9,PSEN1,RAF1,MAPK14
GOTERM_CC	cilium	4	0.0159	AKT1,CACNA1C,ADRB2,HDAC6
GOTERM_CC	centrosome	4	0.0173	PSEN2,ILK,PSEN1,HDAC6
GOTERM_CC	smooth endoplasmic reticulum	2	0.0178	RYR2,PSEN1
GOTERM_CC	microtubule cytoskeleton	3	0.0213	AKT1,ADRB2,HDAC6
GOTERM_CC	aggresome	2	0.029	PSEN1,HDAC6
GOTERM_CC	nucleus	11	0.0307	AR,NOS3,ILK,AKT1,ADRB2,TNNI3K,PSEN1,RAF1,MAPK14,HDAC6,VEGFA
GOTERM_CC	ciliary basal body	3	0.0313	AKT1,ADRB2,HDAC6
GOTERM_CC	early endosome	3	0.032	PSEN2,ADRB2,PSEN1
GOTERM_CC	sarcoplasmic reticulum membrane	2	0.033	RYR2,PLN
GOTERM_CC	endoplasmic reticulum	5	0.0498	PLN,PSEN2,AKT1,PSEN1,VEGFA

Table S4. GO_MF enrichment analysis results

Category	Term	Count	P-Value	User Ids
GOTERM_MF	enzyme binding	7	0.00000104	RYR2,AR,AKT1,ADRB2,RAF1,MAPK14,HDAC6
GOTERM_MF	protein kinase activity	6	0.0000701	FLT1,ILK,AKT1,TNNI3K,RAF1,MAPK14
GOTERM_MF	calmodulin binding	4	0.000754	RYR2,NOS3,AKT1,CACNA1C
GOTERM_MF	ATP binding	7	0.00152	FLT1,ILK,AKT1,ABCC9,TNNI3K,RAF1,MAPK14
GOTERM_MF	ATPase binding	3	0.0028	AR,PLN,PSEN1
GOTERM_MF	kinase activity	5	0.00326	FLT1,AKT1,TNNI3K,RAF1,MAPK14
GOTERM_MF	nucleotide binding	7	0.00367	FLT1,ILK,AKT1,ABCC9,TNNI3K,RAF1,MAPK14
GOTERM_MF	protein serine kinase activity	4	0.00376	AKT1,TNNI3K,RAF1,MAPK14
GOTERM_MF	calcium channel activity	3	0.00423	RYR2,CACNA1C,PSEN1
GOTERM_MF	beta-catenin binding	3	0.00423	AR,PSEN1,HDAC6
GOTERM_MF	ATPase inhibitor activity	2	0.00528	PLN,HDAC6
GOTERM_MF	protein serine/threonine kinase activity	4	0.00568	AKT1,TNNI3K,RAF1,MAPK14
GOTERM_MF	aspartic endopeptidase activity, intramembrane cleaving	2	0.00704	PSEN2,PSEN1
GOTERM_MF	transmembrane transporter binding	3	0.00758	RYR2,PLN,ABCC9
GOTERM_MF	potassium channel activator activity	2	0.00791	AKT1,ABCC9
GOTERM_MF	identical protein binding	6	0.0177	RYR2,PLN,AKT1,ADRB2,RAF1,VEGFA
GOTERM_MF	transferase activity	6	0.0215	FLT1,AKT1,TNNI3K,RAF1,MAPK14,HDAC6
GOTERM_MF	protein binding	17	0.0249	RYR2,FLT1,NOS3,PSEN2,ILK,CACNA1C,TNNI3K,ADRB2,PSEN1,MAPK14,HDAC6,VEGFA,AR,PLN,AKT1,RAF1,DRD3
GOTERM_MF	protein homodimerization activity	4	0.0256	PLN,AKT1,ADRB2,VEGFA
GOTERM_MF	potassium channel regulator activity	2	0.0432	ABCC9,ADRB2

Table S5. KEGGt analysis results

Category	Term	Count	P-Value	User Ids
KEGG	Calcium signaling pathway	7	0.00000221	RYR2,FLT1,PLN,NOS3,CACNA1C,ADRB2,VEGFA
KEGG	VEGF signaling pathway	5	0.00000248	NOS3,AKT1,RAF1,MAPK14,VEGFA
KEGG	Adrenergic signaling in cardiomyocytes	6	0.00000398	RYR2,PLN,AKT1,CACNA1C,ADRB2,MAPK14
KEGG	cGMP-PKG signaling pathway	6	0.00000574	PLN,NOS3,AKT1,CACNA1C,ADRB2,RAF1
KEGG	Chemical carcinogenesis - receptor activation	6	0.0000212	AR,AKT1,CACNA1C,ADRB2,RAF1,VEGFA
KEGG	cAMP signaling pathway	6	0.0000258	RYR2,PLN,AKT1,CACNA1C,ADRB2,RAF1
KEGG	Neurotrophin signaling pathway	5	0.0000393	PSEN2,AKT1,PSEN1,RAF1,MAPK14
KEGG	Relaxin signaling pathway	5	0.0000537	NOS3,AKT1,RAF1,MAPK14,VEGFA
KEGG	MAPK signaling pathway	6	0.0000998	FLT1,AKT1,CACNA1C,RAF1,MAPK14,VEGFA
KEGG	Focal adhesion	5	0.000302	FLT1,ILK,AKT1,RAF1,VEGFA
KEGG	Diabetic cardiomyopathy	5	0.000313	RYR2,PLN,NOS3,AKT1,MAPK14
KEGG	Rap1 signaling pathway	5	0.000356	FLT1,AKT1,RAF1,MAPK14,VEGFA
KEGG	AGE-RAGE signaling pathway in diabetic complications	4	0.000591	NOS3,AKT1,MAPK14,VEGFA
KEGG	HIF-1 signaling pathway	4	0.000759	FLT1,NOS3,AKT1,VEGFA
KEGG	Pathways of neurodegeneration - multiple diseases	6	0.000912	RYR2,PSEN2,CACNA1C,PSEN1,RAF1,MAPK14
KEGG	Growth hormone synthesis, secretion and action	4	0.00103	AKT1,CACNA1C,RAF1,MAPK14
KEGG	Sphingolipid signaling pathway	4	0.00103	NOS3,AKT1,RAF1,MAPK14
KEGG	Dopaminergic synapse	4	0.00132	AKT1,CACNA1C,MAPK14,DRD3
KEGG	Apelin signaling pathway	4	0.00153	RYR2,NOS3,AKT1,RAF1
KEGG	Fluid shear stress and atherosclerosis	4	0.00159	NOS3,AKT1,MAPK14,VEGFA
KEGG	Oxytocin signaling pathway	4	0.00204	RYR2,NOS3,CACNA1C,RAF1
KEGG	PI3K-Akt signaling pathway	5	0.00263	FLT1,NOS3,AKT1,RAF1,VEGFA
KEGG	Alzheimer disease	5	0.00347	PSEN2,AKT1,CACNA1C,PSEN1,RAF1
KEGG	Kaposi sarcoma-associated herpesvirus infection	4	0.00398	AKT1,RAF1,MAPK14,VEGFA

KEGG	Neutrophil extracellular trap formation	4	0.00398	AKT1,RAF1,MAPK14,HDAC6
KEGG	Endometrial cancer	3	0.00431	ILK,AKT1,RAF1
KEGG	Proteoglycans in cancer	4	0.00445	AKT1,RAF1,MAPK14,VEGFA
KEGG	GnRH secretion	3	0.0052	AKT1,CACNA1C,RAF1
KEGG	Fc epsilon RI signaling pathway	3	0.00585	AKT1,RAF1,MAPK14
KEGG	Human cytomegalovirus infection	4	0.006	AKT1,RAF1,MAPK14,VEGFA
KEGG	Chemical carcinogenesis - reactive oxygen species	4	0.006	AKT1,RAF1,MAPK14,VEGFA
KEGG	Renal cell carcinoma	3	0.00601	AKT1,RAF1,VEGFA
KEGG	Prolactin signaling pathway	3	0.00618	AKT1,RAF1,MAPK14
KEGG	Ras signaling pathway	4	0.00684	FLT1,AKT1,RAF1,VEGFA
KEGG	Pancreatic cancer	3	0.00724	AKT1,RAF1,VEGFA
KEGG	EGFR tyrosine kinase inhibitor resistance	3	0.00779	AKT1,RAF1,VEGFA
KEGG	PD-L1 expression and PD-1 checkpoint pathway in cancer	3	0.00978	AKT1,RAF1,MAPK14
KEGG	GnRH signaling pathway	3	0.0104	CACNA1C,RAF1,MAPK14
